# Feasibility study of the assessment of upper limb function in children with Unilateral Cerebral Palsy using an end-effector robotic device

**DOI:** 10.1186/s12984-026-01950-7

**Published:** 2026-03-19

**Authors:** Veronica Barzacchi, Elena Beani, Silvia Filogna, Giada Sgherri, Valentina Menici, Giacomo Marsanich, Stefano Mazzoleni, Giovanni Cioni, Giuseppina Sgandurra

**Affiliations:** 1 Department of Developmental Neuroscience, IRCCS Fondazione Stella Maris, Calambrone, Pisa, Italy; 2 Department of Clinical and Experimental Medicine, University of Pisa, Pisa, Italy; 3 Department of Electrical and Information Engineering, Politecnico di Bari, Bari, Italy; 4 The BioRobotics Institute, Scuola Superiore Sant’Anna, Pisa, Italy; 5 IMT School for Advanced Studies Lucca, Lucca, Italy

**Keywords:** Upper extremity, Child, Cerebral Palsy, Robotics, Technology, Feasibility studies

## Abstract

**Background:**

Children with Unilateral Cerebral Palsy (UCP) are characterized by significant upper limbs (ULs) impairment that hinders daily activities and participation, requiring accurate assessment. Although motor impairment mainly affects one side, significant evidence suggests that the less affected side (LAS) may also present impairments. Considering the growing interest in pediatrics technologies, a complementary approach combining standardized clinical scales and robotic devices may support a more objective and quantitative evaluation of ULs performance with high precision and objectivity. This study aimed to investigate the feasibility of a planar end-effector robotic device in children and adolescents with UCP and to explore whether robotic ULs indices show associations with clinical measures.

**Methods:**

Twenty-eight children and adolescents with UCP (mean ages: 10.90 ± 3.32 years; 8 males, 20 females) underwent a single-session protocol comprising: (i) clinical assessment based on ULs classifications - Manual Ability Classification System (MACS), House Functional Classification System (HFCS) - and clinical standardized scales - Melbourne Assessment 2 (MA2), Box and Block Test (BBT) -; (ii) robotic assessment protocol with MOTORE with more and less affected side (MAS and LAS respectively), and (iii) feasibility questionnaires completed by both children and clinicians to evaluate usability and acceptability. Feasibility data were analysed as raw scores (mean value, SD, range) and percentages. Two different MANOVA analyses were applied to robotic parameters, including limb condition (LAS vs. MAS), hand dominance, and age as predictors, with HFCS or MACS levels analysed in separate models to avoid conceptual overlap and collinearity. Finally, Spearman’s r correlation coefficient assessed correlations between clinical and robotic measures.

**Results:**

Feasibility questionnaires showed positive results, supporting the system’s user-feasible design. Across the MOTORE tasks, MANOVA analyses showed that HFCS or MACS levels and limb severity condition (MAS vs. LAS) were the main factors affecting robotic performance, with age influencing selected parameters, while hand dominance had no significant effect. Finally, correlation analyses further revealed moderate to strong associations between MOTORE’s robotic parameters and clinical scores (r range: 0.375–0.643).

**Conclusions:**

This paper highlights the feasibility of MOTORE robotic system in pediatric assessment setting both for users and clinicians. The observed associations suggest that the system may provide complementary quantitative information related to ULs functions. As these findings are exploratory, further studies are required to establish validity, reliability, and responsiveness before clinical integration of the system to support objective and quantitative data to guide individualized rehabilitation strategies for children with UCP.

*Trial registration*: ClinicalTrial.gov: NCT06012617 and NCT06666829.

**Supplementary Information:**

The online version contains supplementary material available at 10.1186/s12984-026-01950-7.

## Introduction

Cerebral palsy (CP) is the most common motor disability in childhood [1], counting 2–3/1000 live births, up to 40–100/1000 among very premature and very low birth-weight infants [2]. According to the updated 2025 description, CP is an early-onset, lifelong neurodevelopmental condition characterized by activity limitations resulting from impairments in the development of movement and posture [3]. Unilateral CP (UCP), the most frequent form (36% of cases), mainly involves one side of the body, often resulting in greater impairment of UL than the lower limb [4]. It is characterized by spasticity, sensory deficits, and reduced strength [5] with severity depending on the timing, site, extent, and nature of the brain lesion [6]. Children with UCP frequently exhibit a significant impairment in reaching, grasping, releasing and manipulating objects with the more affected side (MAS) (e.g. slow and jerky reaching pattern) [7], critically hindering motor control and execution [8]. Studies have mainly focused on the motor deficits of the MAS, but the dominant limb (which we refer to as less affected side (LAS)) also exhibits impairments in coordination, dexterity and strength [9]. The UL challenges could impact the ability to perform daily activities, which depends on the coordination of both arms, thereby limiting participation in educational, recreational, and later, professional roles. This emphasizes the need for a comprehensive understanding of both ULs [9].

A comprehensive evaluation of UL function in children with UCP is crucial for guiding treatments and monitoring its effectiveness over time [10]. Several clinical measures and classification systems are currently available to assess ULs impairments in this population and can be used in combination for a more comprehensive analysis [11]. Specifically, the Manual Ability Classification System (MACS) [12] classifies how children use their hands in daily activities according to their level of independence, while the House Functional Classification System (HFCS) [13] focuses on the functional use of the more affected hand. In parallel, standardized assessments such as the Melbourne Assessment (MA2), Assisting Hand Assessment (AHA), Shriners Hospital Upper Extremity Evaluation (SHUEE), Quality of Upper Extremity Skills Test (QUEST), and questionnaires as ABILHAND-Kids are recommended to evaluate unimanual capacity, bimanual performance, and functional abilities in children with UCP [11, 14]. However, clinical scales are inherently operator-dependent, which can lead to potential inaccuracies due to the subjective nature of the assessment; moreover, is reported the small sensitivity and high time-consuming in application [15].

In this context, the advent of technology has opened new avenues introducing innovative approaches in this field. Indeed, robotic systems have emerged as promising tools for assessment and rehabilitation also in children, offering repetitive and goal-oriented activities aligned with neuroplasticity principles [16], while promoting sensorimotor and cognitive improvements and enabling quantitative evaluation of kinematic parameters and interaction forces to monitor children’s progress [17]. Robotic devices can be used to evaluate children’s motor functions, in combination with traditional clinical assessments, offering a quantitative and reliable description of motor performance, and an accurate, repeatable and sensitive assessment [18].

Robotic systems can provide high temporal and spatial resolution metrics that allow recording joints position, velocities and interaction forces and extracting parameters [19], thanks to the embedded sensors in the devices, that can provide valuable insights into motor function [19, 20]. Recent studies involving paediatric population have shown the potential to extract kinematic and dynamic measurements directly from robotic data, both during and after rehabilitation training [21–24]. Other studies have specifically used these devices as assessment tools to evaluate ULs sensorimotor functions, independently from the training [25, 26].

Among the various ULs robotic systems, notable examples include InMotion2 [21], Armeo Spring [27], and MOTORE (MObile RoboT for upper Limb NeurOortho REhabilitation) [28]. While the first two systems have already been applied to pediatric populations, MOTORE, an innovative planar end-effector robotic device, has yet to be explored in this population group. This robot has been designed with specific mechanical, electrical, and control solutions tailored to neurorehabilitation, particularly targeting shoulder and elbow movements. MOTORE has been used as a rehabilitative [28] and an evaluation tool [29] in adults, but to our knowledge its feasibility and application in pediatric population have not yet been investigated. Following the recommendation of Sgherri et al. [30] it would be necessary to assess also the feasibility of MOTORE as the success of any developed technology strongly depends on the perspectives and acceptance of both end-users and healthcare professionals, which improve not only the efficiency and safety of the device but also its marketability.

In this framework the primary objective of this study was to investigate the feasibility of using MOTORE as evaluation tool in a group of children with UCP, as the integration of robotic systems into pediatric rehabilitation requires careful consideration of their usability and adaptability to children. Furthermore, as secondary objective, we aimed to explore MOTORE capability to obtain quantitative ULs indices during unassisted reaching tasks, both with MAS and LAS. Specifically, we investigated whether clinical and individual factors influenced global robotic performance by developing two separate multivariate models (i) a first examining the effect of different levels of MAS motor ability, as defined by HFCS, limb severity condition (LAS vs. MAS), age and hand dominance on robotic outcomes; (ii) a second model exploring the effects of functional manual ability, as categorized by the MACS, limb condition, age and hand dominance on robotic performance. Finally, we explored (iii) the statistical relationship between robotic indices and clinical scores.

## Materials and methods

### Participant

Children and adolescents with UCP who had been referred to IRCCS Fondazione Stella Maris (Pisa, Italy) were enrolled according to the following inclusion criteria: (1) confirmed diagnosis of spastic UCP; (2) age between 6 and 18 years; (3) mild to moderately severe ULs impairment with minimal ability to grasp and hold objects with affected hand (MACS level I-III); (4) sufficient cooperation, cognitive and communicative understanding to perform assessments; (5) adequate attention, commitment and visual skill. Exclusion criteria were: (1) orthopedic surgery or an intramuscular botulinum toxin A injection in UL within 6 months prior to enrolment; (2) uncontrolled seizures; (3) behavioral and cognitive disorders and/or reduced compliance that would interfere with assessments; (4) inability to discriminate distinctly the images shown on a 22″ monitor at a distance of about 50 cm, even with corrective glasses. Recruitment was carried out at IRCCS Fondazione Stella Maris. This feasibility study is part of a larger Catch-Hemi and Fit4MedRob studies, approved by the Paediatric Ethics Committee (53/2022 and 151/2024 respectively) and registered on ClinicalTrial.gov (NCT06012617 and NCT06666829 respectively).

### Assessment protocol

Participants underwent an evaluation session consisting of an initial clinical assessment based on UL classifications and administration of clinical scales, followed by an assessment session using the robotic system. All assessments are described in detail in the following paragraphs. At the end of the evaluation, an ad hoc questionnaire was administered to both children and clinicians to investigate feasibility. The entire protocol lasted for a maximum duration of 2 h, for each participant, in a single evaluation session.

### Clinical assessment protocol

Participants were classified according to MACS and modified HFCS. Moreover, they were clinically evaluated using two standardized tests measuring unimanual ability: MA2 and Block and Block Test (BBT). The assessments were carried out by two therapists with experience in children’s assessments and scored by other therapists in a blind way. Scorer were blinded to both the robotic metrics and the characteristics of the children.

#### Manual ability classification system (MACS)

The MACS describes how children with CP handle and manipulate objects in daily activities on a five-level scale, where level I corresponds to the best performance. The child’s usual performance, not the maximal capacity, is classified. It can be used in children from 4 to 18 years. The MACS demonstrates excellent psychometric properties, with very high interrater reliability both between therapists (ICC = 0.97) and between parents and therapists (ICC = 0.96), strong construct validity through correlations with other CP classification systems, and good stability over time, supporting its use as a reliable measure of manual performance in children with CP [12].

#### Modified House Functional Classification System (HFCS)

Modified HFCS consists of nine-point scale describing upper extremity function, ranging from a hand that is not used at all (grade 0), across different levels such as passive assist and active assist, to a hand that is used spontaneously and independently from the other during tasks (grade 8). Modified HFCS is a validated and reliable classification system, demonstrating good interrater reliability and construct validity, and is widely utilized to evaluate hand function levels in children with CP [13].

#### Melbourne Assessment 2 (MA2)

The MA2 is a standardized, criterion-referenced test used to assess UL movement quality in children aged 2.5 to 15 years with neurological impairments [31]. It measures four key aspects: (1) active range of movement (ROM), (2) accuracy, (3) dexterity, and (4) fluency through 14 tasks involving object reaching, grasping, releasing and manipulating. Performances are video-recorded and scored across 30 items, providing separate raw scores for each subscale that can be analysed separately. It predominantly includes ICF concepts within the body function domain (ROM and fluency subscales) as well as in the activity domain (dexterity subscale) or both (accuracy subscale). Although the MA2 is validated for children up to 15 years, it has been used in older individuals [32]. In this study, we have chosen to maintain a consistent assessment across all ages by using the MA2, rather than switching to other scales. The MA2 demonstrates high inter-rater reliability, with ICC values ranging from 0.92 to 0.98, and significant correlations with other standardized UL measures (e.g., BBT, Bruininks-Oseretsky Test of Motor Proficiency, 2nd edition), supporting its validity and responsiveness in children with CP [33].

#### Block and Block Test (BBT)

BBT is a quick, simple, and reliable test of gross unimanual dexterity within the ICF activity domain, suitable for individuals from childhood to adulthood. Using a two-compartment box with 150 wooden blocks, the task involves moving as many blocks as possible in one minute, one at a time, from one compartment to another. Each hand is tested separately, starting with the less affected one. The score is based on the number of blocks moved for each hand. Its user-friendly design and ease of understanding make it particularly suitable for children [34]. The BBT demonstrates good reliability, with ICC values ranging from 0.85 to 0.99, and shows good construct validity, as indicated by significant correlations with the MA2 and other standardized UL measures, supporting its use as a complementary tool to assess manual dexterity in children with CP [33, 35].

### Robotic assessment protocol

#### Robotic device and system configuration

**Fig. 1:**
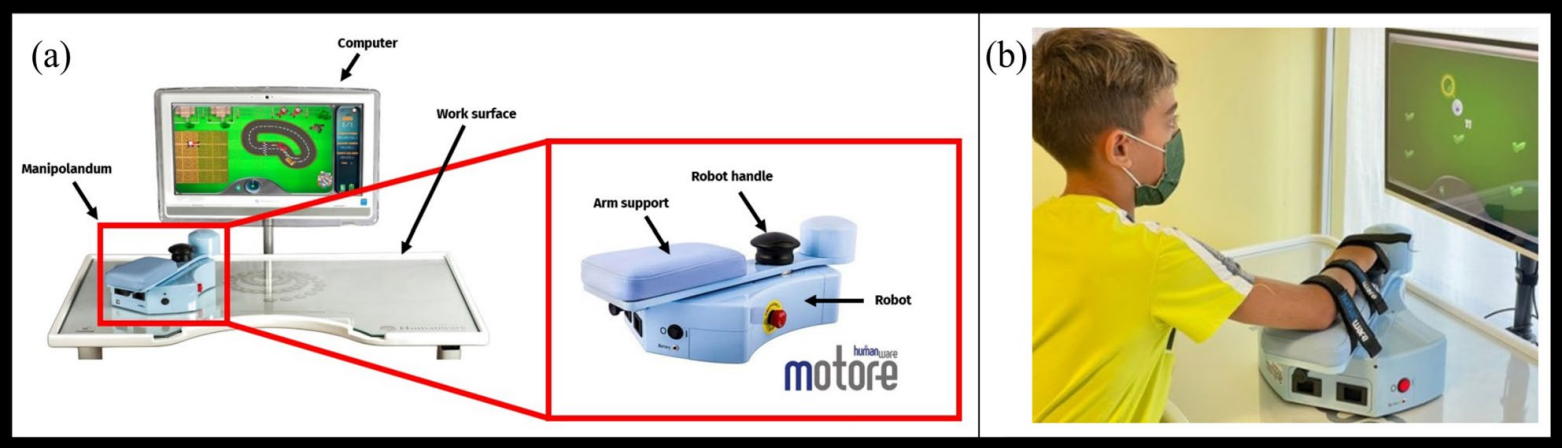
**a** MOTORE; **b** A child who performs an exercise with MOTORE

 The robotic assessment of ULs motor performance was conducted using MOTORE, a CE-marked, class IIa, planar end-effector robotic device (Humanware Srl, Pisa, Italy). The system comprises (i) a mobile robot (called manipulandum) with interchangeable handles, (ii) a working surface with an optical localization system, and (iii) a computer with dedicated software (Fig. 1a). Data were recorded at a sampling frequency of 100 Hz.

Seated at height-adjustable table, users interact with the system by placing their forearm on the manipulandum’s arm support, secured with Velcro^®^ straps, and grasping the handle, which incorporates a two-axis load cell sensor able to detect interaction force direction and magnitude. Users can actively move the manipulandum on the working surface (or be assisted by the robot) through the integrated wheels (Fig. 1b). The optically coded surface enables precise tracking of the manipulandum’s position, while safety edges prevent accidental falls. Exercises are customizable in terms of parameters, complexity, and interaction modes (passive, active, assistive).

Hardware- and software-enforced safety limits are implemented, including a minimum force activation threshold (< 0.5 N), a maximum exerted force of 50 N (automatically limited by the controller), and a maximum velocity of 0.8 m/s (controller-limited), ensuring safe operation during task execution.

Three handle configurations are available to accommodate different user needs: a horizontal spherical grip, a vertical cylindrical (joystick-like) grip, and a wrist handle for users with difficulties in grasping.

#### Sensing architecture and data acquisition

The MOTORE system integrates multiple sensing components for planar kinematics and force measurement. Planar interaction forces between the user’s hand and the manipulandum are measured by a two-axis load cell embedded in the device handle [36]. Manufacturer specifications report a force sensitivity < 0.5 N and a force measurement accuracy of 0.01 N over a full scale of 40 N. At each system powering on, an automatic software-managed calibration procedure is performed to zero the load cell signal.

Planar end-effector position is obtained through an absolute localization system combining (i) odometry derived from motor encoders and (ii) an optical localization subsystem based on Anoto^®^ technology [28, 36]. Encoder- and optical-derived estimates are fused within the device firmware using an Extended Kalman Filter [36]. Manufacturer- reported system-level position accuracy is approximately 0.4 mm.

A detailed technical description of the robotic system, including sensing architecture and signal processing, is provided in Additional file 1 - Technical description of the robotic system.

#### Procedure robotic tasks and indices

Participants performed a protocol consisting of five exercises implemented on the device. For each exercise, task-specific robot settings (e.g., stiffness, viscosity, and other control parameters) were defined in the experimental protocol and kept constant across participants.

The first two exercises (Small ROM and Large ROM), categorized as “evaluative exercises”, required participants to reach targets from the center along eight radial directions. The two exercises differed in the target distance from the center: in the Small ROM, targets were placed closer to the center, whereas in the Large ROM targets were positioned farther away, resulting in a greater spatial extent of the workspace. During these exercises, the robot operated in a free mode, allowing unconstrained planar movement. Participants followed visual arrows to reach each target (marked by a yellow circle), returned to the center between attempts, and completed a total of 16 clockwise reaching movements.

**Fig. 2:**
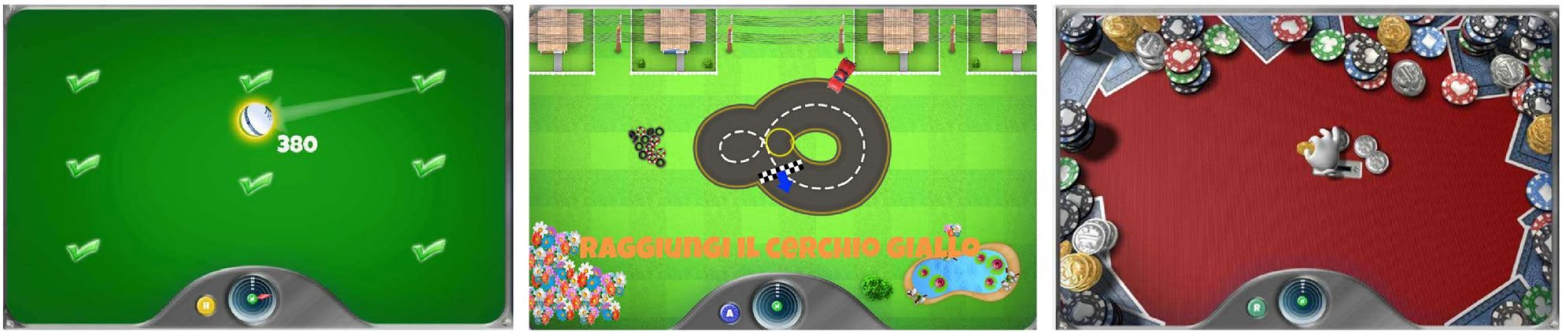
Graphical representation of the MOTORE exercises used in the study. From left to right: ROM task, small 8-Trajectory task, and Coins task

 Subsequently, three exercises selected from “rehabilitation exercises” library were performed Two required moving a virtual car along a constrained 8-shaped trajectory, one small (Small 8-trajectory) and one large (Large 8-trajectory). The third exercise (Coins) consisted of sequentially picking up five coins, one at time, arranged along an arc from the center of the workspace and releasing them into target areas. In these constrained tasks, deviation from the ideal trajectory resulted in increasing resistive forces applied by the robot to guide the participant back toward the reference path. Visual feedback for each exercise is shown in Fig. 2.

Participants performed the tasks seated in front of a height-adjustable table, with the monitor positioned at eye level and the workspace centered at participants midline. Subjects’ forearm was placed on a dedicated support on the device, with their hand grasping the horizontal handle. Each participant completed the exercises first using the LAS and then the MAS. After each trial, a summary report was exported via the dedicated software, as outlined in Table 1. To address the issue of multiple comparisons, primary robotic outcomes were defined a priori. Based on their clinical relevance and consistency across tasks, Area and Work were selected as primary outcomes for ROMs tasks, and Accuracy Error and Velocity were selected as primary outcomes for 8-Trajectory tasks and Coins.

**Table 1:** Summary of robotic parameters extracted from MOTORE across the exercises

Parameter	Description	Equation	Small ROM	Large ROM	Small 8-trajectory	Large 8-trajectory	Coins
Area (m^2^)	Total area covered by the patient, including both correct and incorrect movements.	$A_{{\mathrm{cov} ered}} = \mathop \sum \limits_{{k = 1}}^{{120}} \frac{1}{2}\,r_{{\max }}^{2} \left( {\theta _{k} } \right)\,\Delta \theta$	✓	✓			
Time (m)	Time to complete the exercise.		✓	✓	✓	✓	✓
Work (J)	Patient’s effort to push the manipulandum along the correct trajectory.	$W_{P}^{{tot}} = \mathop \smallint \limits_{0}^{T} \left| {F_{{cell,i}} ~ \cdot ~\hat{u}_{{v,i}} } \right|\parallel u_{{v,i}} \parallel dt$ $W_{P}^{{use}} = \mathop \smallint \limits_{0}^{T} \left( {F_{{cell,i}} ~ \cdot ~\widehat{{t_{i} }}} \right)\parallel u_{{v,i}}^{{\tan }} \parallel dt$	✓	✓	✓	✓	✓
Velocity (%)	Normalized average speed respect to task reference.	$v_{{norm}} = \frac{{\bar{v}_{{user}} }}{{v_{{robot,\max }} }}$	✓	✓	✓	✓	✓
Accuracy error (mm)	Error measured based on the average distance from the ideal trajectory.	$err_{n} = \sqrt {\left( {x_{r} - x_{i} } \right)^{2} + \left( {y_{r} - y_{i} } \right)^{2} }$ $acc_{{err}} = \frac{1}{N}\sum err_{n}$			✓	✓	✓

*For MOTORE parameters*, i.e.,* Area (total area covered*,* including correct and incorrect movements)*,* Time (duration to complete the task)*,* Work (effort to move the manipulandum along the correct trajectory)*,* Velocity (user average speed normalized for predefined task velocity)*,* and Accuracy error (average distance from the ideal trajectory) description and mathematical formulas have been reported. Columns indicate the exercise: Small ROM*,* Large ROM*,* Small 8-trajectory*,* Large 8-trajectory*,* and Coins. A check mark (✓) denotes that the parameter was recorded for the corresponding exercise.*

### Feasibility questionnaire

MOTORE’s feasibility was assessed using an ad-hoc questionnaire inspired by standardized tools and scientific criteria. The domain considered were usability [37–39] and acceptability [40], based respectively on ISO/DIS 9241-11 and the Technology Acceptance Mode (TAM). Following the approach described by Sgherri et al. [30], the questionnaires were specifically tailored to MOTORE, as instruments evaluating usability and acceptability need to be adapted to the technology under investigation.

 The development process involved several steps. Initially, items were drafted based on literature review, ensuring alignment with the targeted domains. Draft versions were then discussed between clinicians and refined according to their feedback to improve clarity, relevance, and appropriateness for children and adolescents. Accordingly, all items were developed specifically for the assessment of MOTORE. This questionnaire has been extensively described in a recent study [41], where the same methodological criteria were applied to investigate feasibility across different technologies. Two versions of the questionnaire were developed: one for the children/adolescents carrying out the robotic assessment protocol and one for the clinicians conducting the session. Both versions included an introductory section on participants’ details, session duration, and whether the system was perceived as a game or therapy. The clinicians’ version comprised 16 questions evaluating usability and acceptability (8 for each domain), while the children’s included 18 questions, adding a motivation section (2 questions) in addition to usability and acceptability. Responses were rated on a 5-point Likert scale (1 most negative, 5 most positive): to facilitate their comprehension, children’s responses used a simplified “smiley-meter” with five emoticons. The total possible score for each section is as follows: usability 8 minimum −40 maximum; acceptability 8 minimum −40 maximum; motivation to use 2 minimum – 10 maximum. Therefore, for clinician’s questionnaire the total score is 80, for children questionnaire is 90. Internal consistency of the questionnaire was assessed using Cronbach’s alpha across items for children and clinicians, yielding values of 0.82 and 0.76, respectively. Questionnaires were completed after each session by clinicians and by participants with adequate cognitive abilities.

### Statistical analysis

Participants demographic and clinical data (sex, age, dominance, classification, and clinical scores) were collected to describe the population sample. Descriptive statistics are reported as mean value and standard deviation (SD) (range: min-max) according to variables’ distribution. Data normality was verified with Shapiro-Wilk’s Test; due to the non-normal distribution of the majority of the data, non-parametric analyses were used. Significance was set at *p* <.05.

For the feasibility questionnaire data, total raw score (mean value, SD, range), as well as totals and relative percentages for each area, were calculated. Data recorded by the robot were extracted from exercise reports and exported as CSV files. Exploratory analysis was conducted using MATLAB (R2024a, The MathWorks, Inc., Natick, Massachusetts, USA) to summarize features and detect outliers using the interquartile range (IQR) criterion; no outliers were detected in the study sample; statistical analyses were performed with SPSS 25.0 (IBM Corporation, Armonk, NY, USA).

An a priori power analysis was conducted using G*Power (version 3.1.9.7). For the MANOVA (global effects), the analysis assumed a significance level of α = 0.05, a statistical power of 0.80, and a medium effect size (f²(V) = 0.25). The analysis indicated a minimum total sample size of 25 participants (actual power = 0.85), based on Pillai’s Trace. Additionally, a separate a priori power analysis was performed to determine the required sample size for detecting a correlation of ρ = 0.53. With the same significance level (α = 0.05, two-tailed) and desired power (0.80), this analysis indicated a minimum of 25 participants necessary to reliably detect the expected effect. To account for a potential dropout rate of approximately 10%, the target sample size was increased to 28 participants.

For each robotic task (Small-ROM, Large-ROM, Small and Large 8-Trajectory, and Coins), two separate MANOVA models were applied using robotic parameters as dependent variables. In the first model, HFCS levels, limb severity condition (MAS vs. LAS), hand dominance, and age (covariate) were included as predictors. In the second model, HFCS was replaced by MACS level, while the remaining predictors remained unchanged. HFCS and MACS were analyzed in separate models to avoid conceptual overlap and potential collinearity. Significant multivariate effects (Pillai’s Trace, *p* <.05) were followed by univariate analyses with false discovery rate (FDR)–adjusted p-values, according to the Benjamini–Hochberg procedure. Effect sizes were reported as partial eta squared (ηp²). Pillai’s Trace was selected as the MANOVA test statistic due to its relative robustness to violations of multivariate normality and homogeneity of covariance matrices [42]. However, we recognize that non-normality may influence the results, and findings should be interpreted with caution. Finally, Spearman’s rank correlation coefficient (r) was calculated to investigate the relations between clinical classification/scores and robotic parameters, categorized as very strong (*r* >.90), high (r 0.70–0.90), moderate (r 0.40–0.70), low (r 0.20–0.40), and slight (*r* <.20) [43].

## Results

### Participants characteristics

A total of 28 children and adolescents (mean age 10.90 ± 3.32 years) participated in the study. Demographic and clinical characteristics are summarized in Table 2. Participants were classified according to MACS and HFCS levels. Since HFCS categorizes upper extremity function as follows: level 0 for no UL use, level 1–3 denote a poor, fair or good passive assist, level 4–6 indicates an UL with poor, fair or good quality of active assist, level 7–8 represent a partial or complete spontaneous use, we grouped the sample into two categories: group 1 (HFCS levels 7–8, *n* = 12) and group 2 (HFCS levels 4–6, *n* = 16), as already used [44]. Clinical results for MA2 and BBT assessment are shown in Table 2.

**Table 2:** Participants' demographic and clinical characteristics

Children with UCP (N=28)			
	Mean ± SD		Range
Age at assessment	10.90 ± 3.32 years		(7.03 - 17.97)

Demographic characteristics			
Characteristic	Category	N	Percentage
Sex	Female	8	(28.6%)
	Male	20	(71.4%)
Dominance	Right	13	(46.4%)
	Left	15	(50.0%)
MACS	Level I	16	(57.1%)
	Level II	12	(42.9%)
HFCS	Level IV	5	(17.8%)
	Level V	8	(28.6%)
	Level VI	3	(10.7%)
	Level VII	8	(28.6%)
	Level VIII	4	(14.3%)
HFCS groups	Group 1	12	(42.9%)
	Group 2	16	(57.1%)

Clinical results			
Clinical Scales	Subtest/Side	Mean ± SD	Range
MA2	ROM	19.54 ± 6.06	(7 ÷ 27)
	Accuracy	22.89 ± 3.34	(13 ÷ 25)
	Dexterity	11.11 ± 3.83	(4 ÷ 16)
	Fluency	13.43 ± 4.26	(6 ÷ 21)
BBT	Less affected side	50.96 ± 12.95	(28 ÷ 75)
	More affected side	29.07 ± 15.21	(4 ÷ 56)

*Age is reported as mean ± SD (range). Demographic characteristics are reported as N (number of participants) and percentages (proportion within each category.) MACS, Manual Ability Classification System; HFCS, House Functional Classification System; HFCS groups, grouping based on HFCS levels. Clinical results are reported as mean ± SD (range). MA2, Melbourne Assessment 2 (subtests: ROM, Accuracy, Dexterity, Fluency); BBT, Box and Block Test.*

### Feasibility results

 All participants (*N* = 28) completed the evaluation protocol and agreed to answer the feasibility questionnaire through an interview, also adding some personal comments. When asked whether they perceived the activity as a game or therapy: 17 children (60.7%) identified it as a game, while 11 (39.3%) considered it as therapy. The clinicians who conducted each session also completed the questionnaire for each child, yielding 28 clinicians responses corresponding to each assessment. Feasibility outcomes were generally positive among both children and clinicians, with detailed raw scores, ranges and percentages summarized in Table 3. Item-level responses for both children and clinicians are reported in Additional File 2, Table S1.

**Table 3:** Feasibility results for children and clinicians

User	Domain	Total scores (mean ± SD)	Range	Percentage
Children	Usability	34.79 ± 3.41	26 ÷ 40	86.97%
	Acceptability	33.86 ± 4.73	19 ÷ 40	84.65%
	Motivation of use	8.00 ± 2.26	2 ÷ 10	80.00%
	Total score	76,64 ± 8.74	54 ÷ 90	85.16%
Clinicians	Usability	35.18 ± 2.40	27 ÷ 39	87.95%
	Acceptability	31.39 ± 2.74	23 ÷ 36	78.47%
	Total score	66.57 ± 4.84	50 ÷ 75	83.21%

*Total scores are reported as mean ± SD, range and percentages, indicating the proportion of the maximum possible score within each domain. For children, the assessed domains were Usability, Acceptability, Motivation of use, and Total score. For clinicians, the assessed domains were Usability, Acceptability, and Total score*

**Fig. 3:**
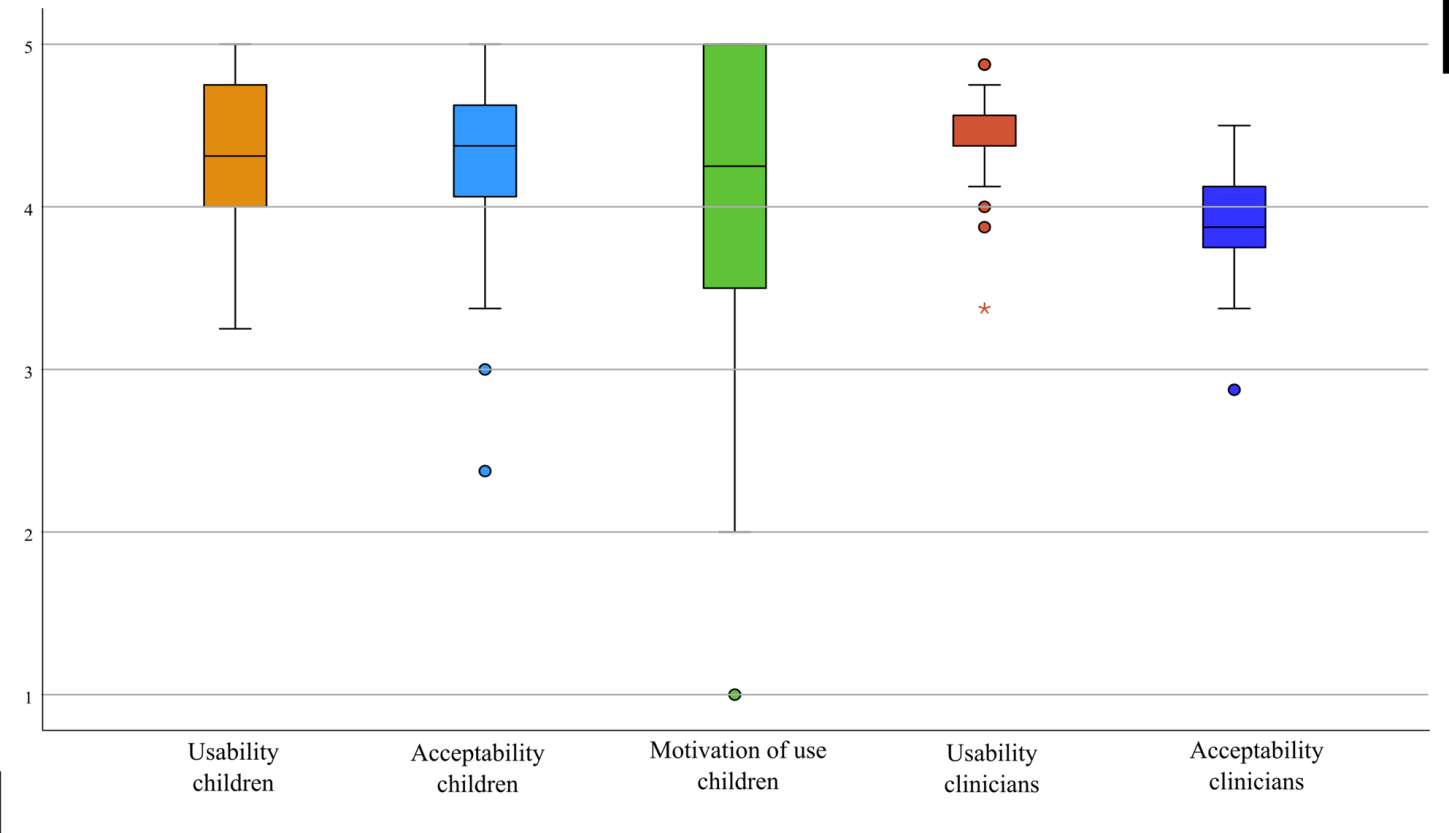
Feasibility questionnaire scores for MOTORE. Boxplots show Likert-scale ratings (1–5) for usability, acceptability, and motivation of use as reported by children, and for usability and acceptability as reported by clinicians. Boxes represent the interquartile range, central lines indicate the median, and whiskers indicate the data range, and individual points represent outliers.

Figure 3 presents the results of the feasibility questionnaire areas, according to Likert scale. Specifically for children, the mean of usability was 4.37 ± 0.96, acceptability 4.24 ± 1.04 and motivation of use 4.03 ± 1.28; for clinicians the mean of usability was 4.35 ± 0.82 and acceptability 3.89 ± 0.91.

### Multivariate analysis of robotic performances across HFCS and MACS levels and limb conditions

 Across the five tasks of MOTORE, the first multivariate analyses consistently identified HFCS levels and limb conditions (MAS vs. LAS) as the primary factors associated with variation in robotic performance. However, MAS and LAS differences may be influenced by the fixed testing order and familiarization effects. Age showed significant multivariate effects in selected tasks. Limb dominance did not contribute significantly. Table S2 in Additional file 3 reports all analyses.

For the small-ROM task, a significant multivariate effect was observed for HFCS (Pillai’s Trace = 0.258, F (4,48) = 4.16, *p* =.006, ηp² = 0.258) and limb (Pillai’s Trace = 0.236, F (4,48) = 3.70, *p* =.010), while age and dominance were not significant. At the univariate level, HFCS significantly influenced covered area (padj = 0.016, ηp² = 0.154), work (padj = 0.039, ηp² = 0.090) and velocity (padj = 0.020, ηp² = 0.122), whereas duration was not influenced. Limb comparisons showed significantly higher velocity (padj = 0.004, ηp² = 0.191) for MAS.

For the large-ROM task, significant multivariate effects were found for HFCS (Pillai’s Trace = 0.343, F (4,48) = 6.27, *p* <.001, ηp² = 0.343), limb (Pillai’s Trace = 0.181, F (4,48) = 2.65, *p* =.045, ηp² = 0.181), and age (Pillai’s Trace = 0.183, F (4,48) = 2.70, *p* =.042, ηp² = 0.183), whereas dominance was not significant. Univariate analyses indicated that HFCS significantly influenced covered area (B = − 0.008, padj < 0.001, ηp² = 0.314) and work (B = − 4.43, padj < 0.001, ηp² = 0.220). Limb differences showed significantly lower covered area (B = − 0.016, padj = 0.008, ηp² = 0.176) for LAS.

For the small 8-trajectory task, significant multivariate effects were observed for HFCS (Pillai’s Trace = 0.253, F (5,47) = 3.18, *p* =.015, ηp² = 0.253) and limb (Pillai’s Trace = 0.298, F (5,47) = 4.00, *p* =.004, ηp² = 0.298), whereas age and dominance were not significant. Limb comparisons revealed significantly lower time execution (B = 1.114, padj = 0.005, ηp² = 0.147), higher accuracy error (B = − 4.607, padj = 0.001, ηp² = 0.203), higher work (B = − 8.149, padj < 0.001, ηp² = 0.253), and higher velocity (B = − 12.000, padj < 0.001, ηp² = 0.222) for MAS.

For the large 8-trajectory task, significant multivariate effects were observed for HFCS (Pillai’s Trace = 0.188, F (4,47) = 2.716, *p* =.041, ηp² = 0.188) and age (Pillai’s Trace = 0.241, F (4,47) = 3.728, *p* =.010, ηp² = 0.241), whereas dominance and limb were not significant. Univariate follow-up analyses did not reveal any significant effects.

For the Coins task, significant multivariate effects were found for HFCS (Pillai’s Trace = 0.225, F(4,48) = 3.481, *p* =.014, ηp² = 0.225), limb (Pillai’s Trace = 0.246, F(4,48) = 3.913, *p* =.008, ηp² = 0.246), and age (Pillai’s Trace = 0.257, F(4,48) = 4.157, *p* =.006, ηp² = 0.257), whereas dominance was not significant. Limb comparisons revealed significantly, higher accuracy error (B = − 2.679, padj = 0.034, ηp² = 0.107), higher velocity (B = − 5.286, padj < 0.037, ηp² = 0.091) for MAS.

 Across the five tasks of MOTORE, the second multivariate analyses consistently identified MACS and limb severity condition (MAS vs. LAS) as the primary factors associated with variation in robotic performance. However, MAS and LAS differences may be influenced by the fixed testing order and familiarization effects. Age showed significant multivariate effects in selected tasks. Limb dominance did not contribute significantly. Table S3 in Additional file 3 reports all analyses.

For the small-ROM task, a significant multivariate effect was observed only for limb (Pillai’s Trace = 0.051, F (4,48) = 3.060, *p* =.025), while MACS, age and dominance were not significant. At the univariate level, limb significantly influenced velocity (padj = 0.008, ηp² = 0.175).

For the large-ROM task, significant multivariate effects were found for MACS (Pillai’s Trace = 0.257, F (4,48) = 4.150, *p* =.006, ηp² = 0.257), and age (Pillai’s Trace = 0.195, F (4,48) = 2.898, *p* =.032, ηp² = 0.195). At the univariate level, MACS significantly influenced area (padj = 0.006, ηp² = 0.156) and work (padj = 0.004, ηp² = 0.190), and limb influenced area (padj = 0.016, ηp² = 0.148).

For the small 8-trajectory task, significant multivariate effects were observed for MACS (Pillai’s Trace = 0.264, F (4,48) = 4.305, *p* =.005, ηp² = 0.264) and limb (Pillai’s Trace = 0.266, F (4,48) = 4.355, *p* =.004, ηp² = 0.266), whereas age and dominance were not significant. At the univariate level limb influenced time (padj = 0.004, ηp² = 0.149), accuracy error (padj = 0.001, ηp² = 0.204), work (padj < 0.000, ηp² = 0.254) and velocity (padj < 0.000, ηp² = 0.223).

For the large 8-trajectory task, significant multivariate effects were observed for MACS (Pillai’s Trace = 0.200, F (4,47) = 2.931, *p* =.030, ηp² = 0.200) and age (Pillai’s Trace = 0.252, F (4,47) = 3.967, *p* =.007, ηp² = 0.252).

For the Coins task, significant multivariate effects were found for MACS (Pillai’s Trace = 0.186, F(4,48) = 2.743, *p* =.039, ηp² = 0.186), age (Pillai’s Trace = 0.256, F(4,48) = 4.128, *p* =.006, ηp² = 0.256), and limb (Pillai’s Trace = 0.246, F(4,48) = 3.906, *p* =.008, ηp² = 0.246). At the univariate level limb influenced accuracy error (padj = 0.038, ηp² = 0.104) and velocity (padj = 0.039, ηp² = 0.090).

**Fig. 4:**
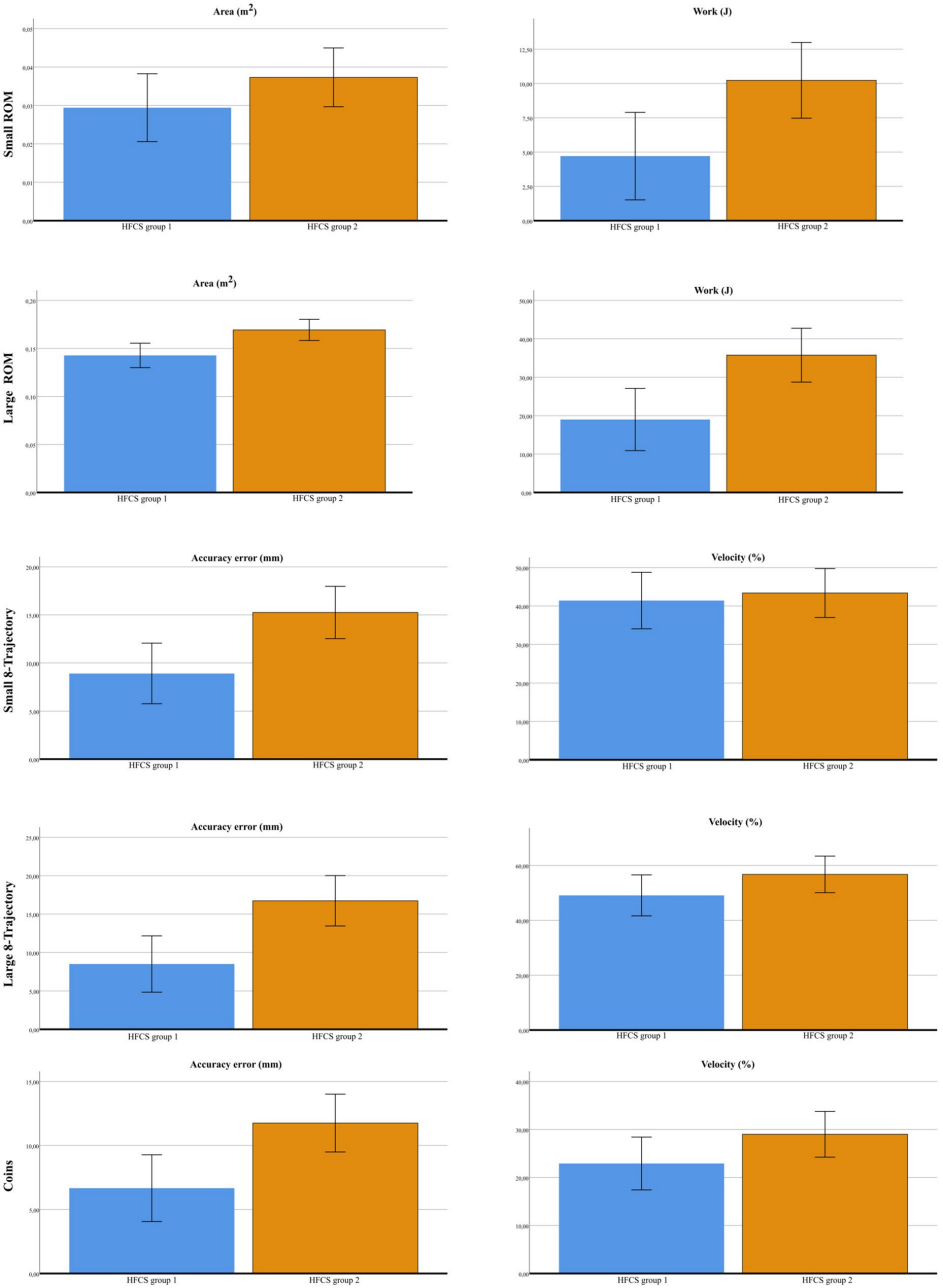
The bar graph shows the mean values and SD of the two HFCS groups (HFCS group 1: N=12; HFCS group 2: N=16) of the main outcomes defined a priori., Specifically are reported Area (m^2^) and Work (J) for Small and Large ROM, and Accuracy error (mm)and Velocity (%) for Small 8-Trajectory, Large 8-Trajectory, and Coins

**Fig. 5:**
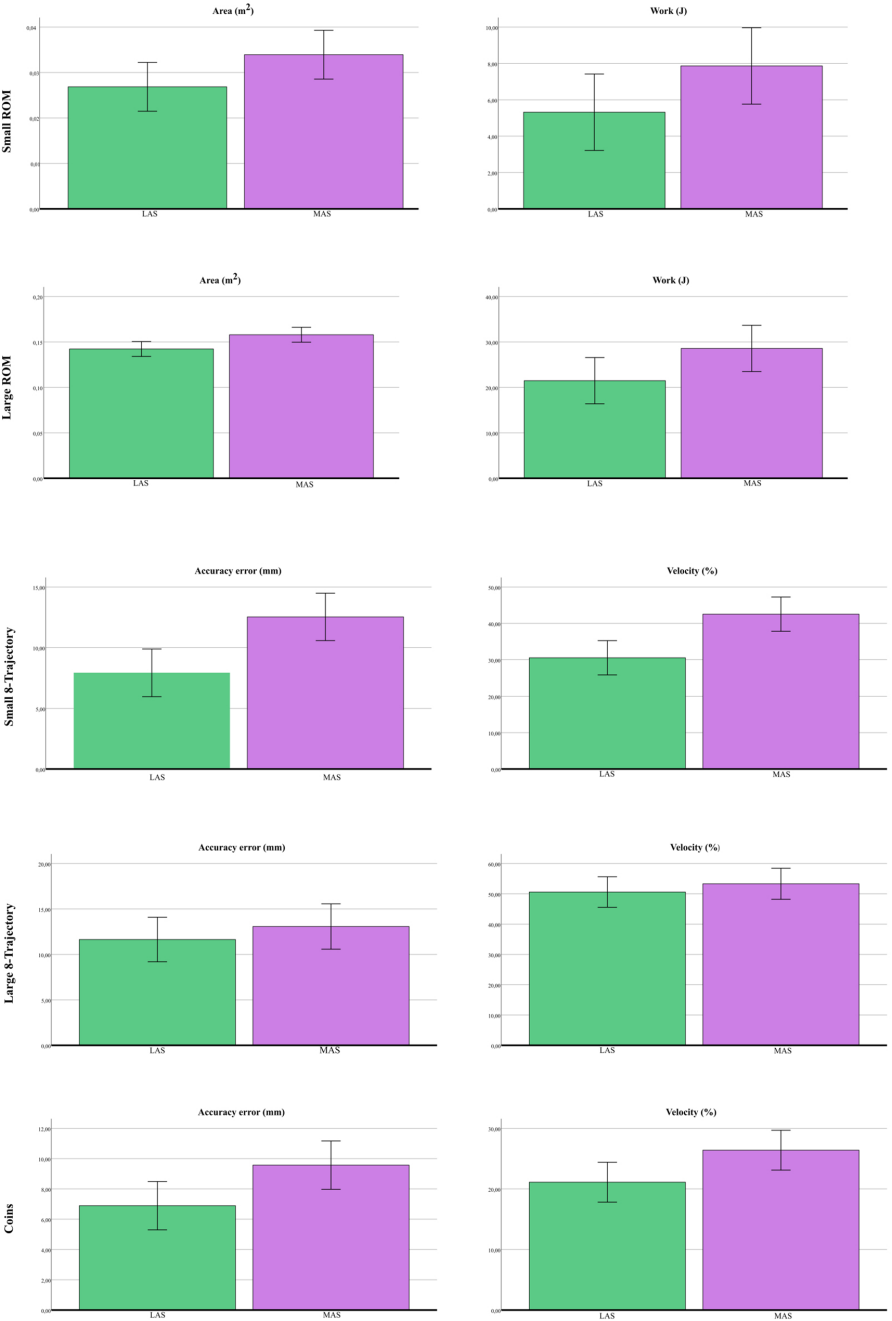
The bar graph shows the mean values and SD of the Least Affected Side (LAS) and More Affected Side (MAS) (N=28 for each group) of the main outcomes defined a priori. Specifically, Area (m^2^) and Work (J) for Small and Large ROM, and Accuracy error (mm) and Velocity (%) for Small 8-Trajectory, Large 8-Trajectory, and Coins

 Figures 4 and 5 show the mean ± SD of the main robotic outcomes defined a priori. Specifically, Fig. 4 reports values for the two HFCS groups (HFCS group 1: *n* = 12; HFCS group 2: *n* = 16), while Fig. 5 compares values between LAS and MAS (LAS: *n* = 28; MAS: *n* = 28). Graphical representations of all robotic indices are provided in the Additional files 4 and 5. Bar plots (mean ± SD) were used for visualization purposes to facilitate comparison with previous robotic studies; however, given the non-normal distribution of several variables, these plots should be interpreted descriptively.

### Correlations between clinical outcomes and robotic indices

The correlation analyses revealed a fair to moderate relationship (r range: 0.375–0.643) between clinical classifications and scores and robotic parameters, as detailed in Table 4.

HFCS exhibited moderate negative correlation between Area and Work for both Small and Large ROM exercises, as well as between Velocity in Small ROM and Time in Large ROM. Additionally, HFCS demonstrated a moderate negative correlation with Accuracy Error and Work of Small and Large 8-Trajectory and Coins tasks, suggesting an association between higher UL impairment levels and changes in robotic parameters related to energy expenditure and movement accuracy. HFCS group correlations were consistent with the pattern observed in the previous findings, except for Area of Small ROM, and Time of Large ROM.

MACS showed positive correlations with Work across the Small ROM, Large ROM, and both 8-Trajectory tasks, indicating an association between higher motor impairment and increased robotic work values. Moreover, Area in the Large ROM and Accuracy error in both the Small and Large 8-Trajectory tasks also demonstrated positive correlations with MACS.

Overall, MA2 subscales showed negative associations with robotic parameters. Regarding ROM exercises the correlation were observed with (i) Area, except for MA2 ROM with Small ROM, (ii) Work, except for MA2 Fluency in Large ROM, and (iii) Time of Large ROM. For the 8-Trajectory exercises the MA2 subscales were correlated with (iv) Accuracy error, except for MA2 Fluency with large 8-trajectory, and with (v) Work, except with MA2 Dexterity subscale with small scenario, and with Fluency subscale with large scenario.

Finally, BBT score of the MAS showed negative associations with multiple robotic parameters, including Area and Work of both Small and Large ROM, Time of Large ROM, and Accuracy error of both Small and Large 8-Trajectory.

**Table 4:** Non-parametric correlation between clinical classifications systems and scores and robotic parameters

		Small ROM				Large ROM				Small 8-Trajectory				Large 8-Trajectory				Coins			
		Area (m^2^)	Time (m)	Work (J)	Velocity (%)	Area (m^2^)	Time (m)	Work (J)	Velocity (%)	Time (m)	Accuracy error (mm)	Work (J)	Velocity (%)	Time (m)	Accuracy error (mm)	Work (J)	Velocity (%)	Time (m)	Accuracy error (mm)	Work (J)	Velocity (%)
HFCS		-,519**	−0,315	-,630**	**-**,414*	-,583**	-,392*	-,581**	0,089	-0,334	-,635**	-,406*	0,154	-0,096	-,543**	-,481*	-0,212	-0,057	-,445*	-,383*	-0,153
HFCS groups		0,291	0,133	,590**	,436*	,545**	0,239	,612**	0,103	0,179	,643**	,572**	0,040	-0,010	,576**	,507**	0,307	-0,101	,512**	,491**	0,322
MACS		0,323	0,345	,500**	0,184	,465*	0,361	,514**	-0,143	0,254	,629**	,482**	-0,157	0,084	,568**	,503**	0,150	0,247	0,332	0,241	0,000
MA2 - ROM		-0,319	-0,281	-,548**	-0,231	-,517**	-,435*	-,468*	0,188	-0,286	-,585**	-,400*	0,111	-0,177	-,427*	-0,366	-0,117	-0,030	-0,318	-0,294	-0,121
MA2 - Accuracy		-,375*	-0,348	-,442*	-0,127	-,435*	-,417*	-,422*	0,205	-0,230	-,508**	-,375*	0,161	0,014	-,405*	-0,336	-0,210	-0,150	-0,246	-0,229	0,015
MA2 - Dexterity		-,376*	-0,311	-,473*	-0,224	-,504**	-,455*	-,410*	0,242	-0,292	-,500**	-0,328	0,109	-0,125	-,404*	-0,378	-0,171	-0,086	-0,325	-0,283	-0,097
MA2 - Fluency		-,395*	-0,277	-,500**	-0,283	-,423*	-,395*	-0,337	0,227	-0,241	-,480**	-,381*	0,105	-0,080	-0,381	-,387*	-0,217	-0,119	-0,234	-0,137	-0,019
BBT – MAS		-,434*	-0,374	-,580**	-0,207	-,582**	-,493**	-,467*	0,227	-0,333	-,510**	-0,334	0,157	-0,314	-,397*	-0,331	-0,065	-0,058	-0,325	-0,328	-0,111

*HFCS* House Functional Classification System; *MACS* Manual Ability Classification System; *MA2* Melbourne Assessment 2; *BBT* Box and Block Test. Positive and negative values indicate direct and inverse correlations, respectively. *p < 0.05; **p < 0.01.

## Discussion

In this study, we explored the feasibility of MOTORE in children and adolescents with UCP and its potential to provide quantitative robotic measures of ULs performance. Overall, the system was well-accepted by both children and clinicians, with high engagement. Key findings of our study highlight the influence of clinical severity and functional classification, particularly HFCS levels than MACS levels, on children’s performance with the robot, as well as differences between MAS and LAS. In addition, significant associations were observed between robotic indices and clinical classifications and scales (HFCS, MACS, MA2, BBT).

This is the first study to explore the feasibility of MOTORE in children and adolescents with UCP, highlighting a significant step forward in the application of robotic devices in pediatric clinical settings. Demonstrating feasibility within the target population is a crucial initial step for implementing an innovative device into clinical practice. Our sample reflects typical demographics of children with UCP [12], with a high representation of children with MACS levels I and II. Since MACS levels stabilize by age 7, the predominance of participants in our group aged 7–11 does not affect the results [45]; in particular, children in MACS levels I, II and III reached 90% of the expected limit at 3, 4 and 8 years respectively [46]. All participants successfully completed the evaluation protocol and the questionnaire’s results indicated satisfactory usability and acceptability scores (> 4 on a 5-point scale for almost all areas). The higher scores were particularly among children who perceived MOTORE as a game—highlighting its strong engagement potential, a key factor in pediatric settings. As expected, and consistent with similar studies [47], patients rated overall their experience with MOTORE more positively than clinicians, likely because the latter tends to be more focused on clinical aims and the correct functioning of the system. While clinicians generally found MOTORE easy to use and effective, they expressed slight concerns regarding its clinical integration (acceptability scores, though positive, were slightly lower). As noted in the literature, fear of innovation, data privacy concerns [48], and resistance to technology adoption [49] are rather common and can influence the overall acceptability of a system. Our findings may reflect the need for some hardware and software modifications to better suit pediatric populations, since MOTORE was originally designed for adult rehabilitation. This could improve the system’s perceived usefulness by aligning the system with clinical objectives, which should, in turn, increase perceived acceptability. Clinicians’ critical perspectives are valuable, highlighting areas for improvement not perceived by children, who focused on the interactive experience. Overall, the system was considered acceptable, featuring intuitive instructions requiring no external technical support, underscoring its user-friendliness and practical applicability in clinical settings. Responses in the Motivation of Use section showed high variability, possibly due to the limited number of questions. Results suggest that the system’s approach could be enhanced through improved personalization (i.e. tailoring game scenarios and feedback), while maintaining standardized evaluation criteria, crucial for comparisons between patients.

Furthermore, to our best knowledge, the present study is the first to examine the potential of MOTORE to obtain quantitative data about the ULs of children. These findings suggest the potential to complement clinical assessment with quantitative descriptors of UL performance in children with UCP.

Two separate multivariate analyses were developed to address complementary but conceptually distinct clinical questions. The first model investigated how HFCS level, together with limb severity condition, age and dominance, influence children’s performance with the robot. The second model explored the association between robotic metrics and manual ability in daily life by using MACS as clinical classification. HFCS and MACS were not included in the same model because, despite being correlated [50], they describe different constructs: HFCS primarily captures unimanual functional capability [13], whereas MACS describes functional autonomy in activities of daily living [12]. Including both variables in the same model would have introduced conceptual overlap and potential collinearity, thus reducing the interpretability of the effects. Overall, the results showed a stronger and more consistent impact of HFCS compared to MACS on robotic performance. This finding was largely expected, given that MOTORE tasks are unimanual. Conversely, MACS classifies functional independence in daily activities, which are influenced not only by motor capacity but also by compensatory strategies, environmental adaptations, and task organization [12]. Nevertheless, the fact that MACS still showed significant multivariate effects in several tasks suggests potential clinical relevance of robotic metrics beyond unimanual capability and represents an exploratory step toward investigating potential links between quantitative robotic outcomes and real-life functioning, an aspect still rarely explored in robot-assisted rehabilitation research [51].

In small-ROM tasks, HFCS significantly influenced global performance. In fact, children with lower HFCS levels showed higher velocity, covered a wider area and showed greater work values. This pattern suggests reduced precision, less linear paths to reach distant targets and more corrective actions, which is consistent with reduced trajectory straightness [52]. Similarly, Bagesteiro [53] observed that children with CP tend to cover greater distances during reaching tasks than typically developing peers, likely due to difficulty in executing direct, precise movements. Higher work values in both types of ROM tasks for children with lower HFCS levels indicate greater effort, resulting in higher energy expenditure. This pattern may also reflect cognitive–motor interference, where managing concurrent task demands can further reduce accuracy in children with higher motor impairment, as shown in dual-task studies in children with CP [54].

In large-ROM tasks, wider movement areas and higher work values were observed, with both HFCS and MACS significantly influencing overall and univariate robotic performance. These tasks are characterized by greater joint excursions and coordination demands, and appear sensitive to impairment severity as well as functional ability as they amplify limitations relevant to everyday UL use [53, 55]. In this context, the emergence of MACS effects may reflect differences in movement quality and efficiency between functional levels: although children with MACS level II are generally independent, their performance is often characterized by reduced speed and quality, with some activities being executed with greater difficulty than children with MACS level I [12]. This functional profile is consistent with the higher effort and reduced accuracy observed in large-ROM exercises and may reflect the use of compensatory strategies during daily bimanual activities.

Across both Trajectory-based exercises and the Coins task, HFCS and MACS showed significant multivariate effects despite the lack of univariate differences. This suggests that clinical classifications mainly affect the overall movement profile rather than single robotic parameters, influencing the multidimensional organization of motor behavior. In complex tasks, relevant impairments may therefore emerge only when performance is considered globally, underscoring the value of multivariate approaches when analysing robotic performance patterns [51, 56].

Limb condition showed a consistent effect across several tasks, particularly in the small-ROM, small 8-Trajectory and Coins exercises. However, LAS and MAS differences should be interpreted with caution, as a fixed test order may have introduced learning or familiarization effects, In ROM tasks, significant differences between ULs emerged in models including both HFCS and MACS, with the MAS exhibiting higher movement velocity in small-ROM tasks and covering a larger movement area in large-ROM tasks. These differences highlight the MAS’s struggle with efficiency and motor control, leading to more dispersive movement patterns with increased variability [56, 57]. In line with motor control frameworks, skilled performance is generally associated with reduced movement variability and more efficient organization of motor behavior [58]. Across tasks requiring precision and continuous control, such as the small 8-Trajectory and Coins exercises, the MAS generally showed higher movement velocity and greater accuracy error, suggesting a tendency to prioritize speed over accuracy and exhibit less accurate movements. This pattern is consistent with Fernani et al.‘s findings [8], who emphasized the need to balance speed and strength with accuracy during therapy. Additionally, higher work values observed in the small 8-Trajectory task indicate increased physical effort, contrasting with principles of motor efficiency that aim to minimize effort while optimizing task performance [59]. Notably, limb-related differences were more pronounced in the small 8-Trajectory task across both models, supporting its sensitivity to deficits in motor control and coordination. In contrast, no significant differences between ULs were observed in the large 8-Trajectory task, likely due to its higher complexity. The task requires reaching distant targets, which may have similarly challenged both limbs and led to compensatory trunk movements [55, 60], reducing the task’s discriminative capacity.

Age showed significant effects, mainly in tasks with higher biomechanical and coordination demands such as large-ROM, large 8-Trajectory, and Coins. This finding is consistent with literature indicating that age plays a role in reaching maturation and UL motor control in children with CP, albeit following a slower and atypical developmental trajectory compared to typically developing peers [52, 61]. These results further support the need to systematically consider age when interpreting robotic metrics in pediatric populations.

A relevant contribution of this study lies in the attempt to bridge robotic assessment with functional classifications of daily life performance. While previous literature has highlighted the difficulty in identifying kinematic or robotic measures that reflect functional outcomes or participation-level domains, the significant associations observed with MACS suggest that robotic metrics may be associated with aspects of motor behavior that are meaningful for everyday functioning [51]. While MOTORE has been validated in adult stroke patients [29], its use in pediatric populations remains underexplored. Our study identified significant associations between MOTORE’s robotic parameters and clinical scores assessing UL function in children with UCP, aligning with a recent systematic review [51] recommending integration of kinematic metrics with clinical scales for comprehensive assessment. The observed correlation patterns highlight robotic tasks and indices that may be particularly informative and clinically interpretable for pediatric UL assessment from an exploratory perspective. Indeed, robotic metrics offer additional quantitative insight that complements, rather than replaces, standard clinical assessments. Specifically, the area and work parameters emerged as the most consistent indices during both types of ROM tasks but particularly in the large ROM task. Moreover, during rehabilitative exercises, accuracy error, together with work, appeared to be the most informative parameters, likely reflecting the child’s ability to control goal-directed movements under task-specific corrective constraints.

Moderate correlations were found between robotic indices (area, work, velocity, time in ROM tasks; accuracy error and work in rehabilitative tasks) and both clinical scales and classifications (HFCS score and HFCS group classification). Weaker correlations with MACS may result from its focus on bimanual independence in daily life, whereas HFCS targets unimanual function. These patterns reflect group differences discussed earlier. Significant correlations were found between MOTORE parameters and MA2 subscales, particularly with area in both ROM tasks—except for the MA2 ROM subscale and small ROM area—suggesting that compensatory strategies (e.g., shoulder/elbow adjustments) seem to have greater impact on distant rather than near targets. Furthermore, significant correlations with other MA2 subscale scores indicate that children with better quality of movement are able to cover less area, expend less effort, and complete tasks more quickly only during large ROM tasks. For rehabilitative exercises, we observe correlations with accuracy errors for both small and large trajectory tasks, except for MA2 Fluency and Large-8 Trajectory, and only some indices reflecting effort. Notably, no significant correlations were found for the Coins task, possibly due to the difficulty of the upward movements required from the center of the workspace. These findings align with previous studies although differences in tasks and kinematic measurement methods complicate the comparisons. Kuczynski and colleagues [26] and Cimolin et al. [27] reported similar relationships between MA2 scores and robotic indices, especially for time and accuracy. Furthermore, movement accuracy is a parameter investigated in studies with adult patients, where strong correlations with many clinical scales are reported [62]. Moderate correlations were also observed with BBT, widely used in the pediatric field [63], particularly in ROM tasks, consistent with its established validity in children with UCP on several clinical measures [34]. Interestingly, more correlations emerged in ROM than in rehabilitative tasks, especially in the large ROM task, likely due to the increased challenge of reaching distant targets. The lower number of correlations with rehabilitative tasks may relate to their inherent corrective nature. Since clinical scales assess both proximal and distal UL functions, the observed partial correlations with MOTORE were expected given that it targets proximal joints. However, robotic systems provide different movement characteristics (such as smoothness) that can be difficult to capture with traditional assessment tools. Therefore, an integrative approach combining robotic and clinical evaluations would be preferable [50]. In this perspective, robotic measures should be considered complementary and adjunctive, providing additional quantitative information that support, rather than replace, traditional clinical assessment.

A key strength of this study is its focus on linking unimanual robotic assessments to functional ULs use in daily life, addressing a gap often overlooked in highly standardized unimanual tasks, as noted by a recent systematic review [64]. Future research should further explore this link to improve real-world applicability. Differences in robotic performance remained meaningful even when considering MACS levels, with the largest effects observed in primarily evaluative ROM exercises compared to rehabilitative exercises used for assessment, suggesting that task type influences performances.

The use of game-like exercises was effective in maintaining engagement and feasibility in children, promoting motivation, compliance, and active participation, factors of particular importance in pediatric populations [65]. Although game-based exercises may not fully replicate real-world functional activities, growing evidence suggests that task-oriented game therapy can also promote improvements in functional performance in daily life in children with CP [66]. However, future studies should include tasks that are more representative of daily activities. Moreover, further investigations are needed to better understand the compensatory strategies often used by children with UCP, such as trunk movements [55, 60], which were not corrected in this study to avoid frustration. Investigating trunk positioning and integrating anthropometric data may offer deeper insight, while personalized rehabilitation approaches (i.e. adapting the robot handle according to the UL ability) could account for the high variability in reaching among children with CP [53].

Another limitation was the fixed order of evaluation (LAS before MAS), which may have unintentionally served as an implicit demonstration, possibly influencing MAS performance due to the novelty effect. As Germanotta et al. [29] suggested, including familiarization sessions could help mitigate this bias and improve assessment accuracy. Potential learning effects were not assessed and should be addressed in future studies that will aim to randomize or counterbalance limb testing order to mitigate potential learning effect. The small sample size limits statistical power and generalizability, and the absence of a control group prevents comparison across populations with different motor impairments.

An additional limitation is the inability to report formal validity and reliability scores for MOTORE system in the pediatric population; in particular, the protocol did not include test–retest reliability and measurement error indices (e.g., ICC, SEM, MDC). While MOTORE has been validated in adult populations [29], its psychometric properties cannot be directly generalized to pediatric populations. The present study was conceived as a first step to investigate clinical feasibility and exploratory construct validity in children; therefore, findings should be interpreted as exploratory and hypothesis-generating and cannot support clinical decision-making or longitudinal monitoring. Because the experimental protocol did not include repeated executions of identical tasks within or across sessions, it was not methodologically feasible to estimate within-session repeatability or between-session test–retest reliability. Future studies should therefore include repeated trials, which would enable the assessment of pediatric validity, reliability, and measurement error. Particularly, reliability studies are a necessary next step before clinical adoption, and the robotic indices presented should currently be considered research tools only. This would, in turn, improve the interpretability of robotic metrics from a clinical perspective.

## Conclusions

This study demonstrated the feasibility and capability of MOTORE to provide valuable insights in a group of children and adolescents with UCP. Feasibility results support the use of this robotic device as clinicians were able to effectively carry out the assessment objectives, while children experienced the sessions as engaging playful activity. This suggests the potential role of robotic systems as complementary tools within pediatric assessment and rehabilitation practices. The significant influence of HFCS levels has provided preliminary evidence of differences in robotic performance across different levels of UL functioning, emphasizing the importance for personalized approaches. The observed association with MACS further suggest a potential relationship between robotic performance and functional abilities in children’s daily life, an aspect that has been insufficiently explored in previous robotic studies. Additionally, the moderate correlations between robotic and clinical indices support further investigation of integratied robotic and clinical assessment approaches. However, these findings should be considered exploratory. Further studies are required to address current limitations before the introduction of MOTORE in paediatric clinical settings. The adoption of more personalized assessment methodologies could maximize the potential of this innovative technology.

## Supplementary Information

Supplementary Material 1.

Supplementary Material 2.

Supplementary Material 3.

Supplementary Material 4.

Supplementary Material 5.

## Data Availability

The anonymized datasets used and/or analysed during the current study are available in the Zenodo repository at 10.5281/zenodo.18044592 link.
