# Supplementary material for: Feasibility study of the assessment of upper limb function in children with Unilateral Cerebral Palsy using an end-effector robotic device
Source: J Neuroeng Rehabil. 2026 Mar 19;23:143. doi: 10.1186/s12984-026-01950-7 (PMC13123123; doi:10.1186/s12984-026-01950-7)
Supplement: Supplementary file 1 — Supplementary Material 1. [file 12984_2026_1950_MOESM1_ESM.docx]

**Additional file 2 – Table S1 - Item-level responses of the feasibility questionnaire**

| **CHILDREN** | **USABILITY** | | | | | | | | **ACCEPTABILITY** | | | | | | | | **MOTIVATION OF USE** | |
| --- | --- | --- | --- | --- | --- | --- | --- | --- | --- | --- | --- | --- | --- | --- | --- | --- | --- | --- |
|  | item1 | item2 | item3 | item4 | item5 | item6 | item7 | item8 | item1 | item2 | item3 | item4 | item5 | item6 | item7 | item8 | item1 | item2 |
|  | 4,39±0,69 | 4,54±0,74 | 4,04±1,07 | 3,75±1,35 | 4,89±0,31 | 3,46±1,10 | 4,82±0,48 | 4,89±0,31 | 4,00±1,12 | 4,57±0,74 | 3,89±1,40 | 4,29±0,94 | 4,50±0,64 | 4,36±0,95 | 4,39±0,74 | 3,86±1,38 | 4,29±1,05 | 3,71±1,49 |
| **CLINICIANS** | **USABILITY** | | | | | | | | **ACCEPTABILITY** | | | | | | | |  |  |
|  | item1 | item2 | item3 | item4 | item5 | item6 | item7 | item8 | item1 | item2 | item3 | item4 | item5 | item6 | item7 | item8 |  |  |
|  | 4,50±0,75 | 4,43±0,57 | 4,18±0,61 | 3,86±0,85 | 3,71±0,90 | 4,75±0,59 | 4,93±0,26 | 4,82±0,39 | 4,54±0,74 | 4,36±0,56 | 3,68±0,72 | 5,00±0,00 | 3,36±0,83 | 3,32±0,90 | 3,82±0,61 | 3,32±0,55 |  |  |

Item-level responses for the feasibility questionnaire administered to children and clinicians. Values are reported as mean ± standard deviation (SD) for each item within the usability, acceptability, and motivation of use domains (the latter assessed in children only).
