# Supplementary material for: Feasibility study of the assessment of upper limb function in children with Unilateral Cerebral Palsy using an end-effector robotic device
Source: J Neuroeng Rehabil. 2026 Mar 19;23:143. doi: 10.1186/s12984-026-01950-7 (PMC13123123; doi:10.1186/s12984-026-01950-7)
Supplement: Supplementary file 2 — Supplementary Material 2. [file 12984_2026_1950_MOESM2_ESM.docx]

**Additional file 3**

**Table S2 - MANOVA results for robotic parameters across tasks according to HFCS levels and limb condition**

|  | **Outcome** | **HFCS 1 Mean ± SD** | **HFCS 2 Mean ± SD** | **β (SE) HFCS1 vs HFCS2** | **t** | **p** | **p_adj** | **95% CI** | **ηp²** | **LAS Mean ± SD** | **MAS Mean ± SD** | **β (SE) LAS vs MAS** | **t** | **p** | **p_adj** | **95% CI** | **ηp²** |
| --- | --- | --- | --- | --- | --- | --- | --- | --- | --- | --- | --- | --- | --- | --- | --- | --- | --- |
| Small ROM | Area (m^2^) | 0.029 ± 0.008 | 0.037 ± 0.019 | −0.004 (0.001) | −3.047 | ***0.004*** | ***0.016*** | [−0.007; −0.001] | 0.154 | 0.027 ± 0.013 | 0.034 ± 0.015 | −0.007 (0.004) | −2.007 | ***0.050*** | 0.100 | [−0.014; 0.000] | 0.073 |
|  | Time (m) | 0.600 ± 0.104 | 0.644 ± 0.167 | −0.015 (0.015) | −0.955 | 0.344 | 0.344 | [−0.045; −0.016] | 0.018 | 0.657 ± 0.169 | 0.625 ± 0.143 | 0.032 (0.041) | 0.777 | 0.441 | 0.441 | [−0.051; 0.115] | 0.012 |
|  | Work (J) | 4.706 ± 1.555 | 10.231 ± 6.948 | −1.198 (0.534) | −2.242 | ***0.029*** | ***0.039*** | [−2.271; −0.125] | 0.090 | 5.32 ± 5.10 | 7.86 ± 5.96 | −2.55 (1.45) | −1.759 | 0.085 | 0.113 | [−5.45; 0.36] | 0.057 |
|  | Velocity (%) | 20.17 ± 3.271 | 24.46 ± 6.025 | −1.305 (0.490) | −2.662 | ***0.010*** | ***0.020*** | [−2.289; −0.321] | 0.122 | 17.86 ± 5.13 | 22.46 ± 5.35 | −4.61 (1.33) | −3.469 | ***0.001*** | ***0.004*** | [−7.27; −1.94] | 0.191 |
| Large ROM | Area (m^2^) | 0.143 ± 0.020 | 0.169 ± 0.225 | −0.008 (0.002) | −4.832 | ***<0.001*** | ***<0.001*** | [−0.012; −0.005] | 0.314 | 0.142 ± 0.018 | 0.158 ± 0.025 | −0.016 (0.005) | −3.298 | ***0.002*** | ***0.008*** | [−0.025; −0.006] | 0.176 |
|  | Time (m) | 0.892 ± 0.219 | 1.087 ± 0.395 | −0.049 (0.028) | −1.747 | 0.087 | 0.116 | [−1.105; 0.007] | 0.056 | 0.971 ± 0.257 | 1.004 ± 0.340 | −0.032 (0.076) | −0.424 | 0.673 | 0.673 | [−0.184; 0.120] | 0.004 |
|  | Work (J) | 18.998 ± 6.426 | 35.756 ± 17.101 | −4.433 (1.170) | −3.790 | ***<0.001*** | ***<0.001*** | [−6.782; −2.085] | 0.220 | 21.48 ± 10.50 | 28.57 ± 15.83 | −7.095 (3.169) | −2.239 | ***0.030*** | 0.060 | [−13.457; −0.733] | 0.089 |
|  | Velocity (%) | 33.750 ± 6.511 | 35.813 ± 8.304 | −0.735 (0.734) | −1.002 | 0.321 | 0.321 | [−2.208; 0.783] | 0.019 | 32.04 ± 8.08 | 34.93 ± 7.53 | −2.893 (1.988) | −1.455 | 0.152 | 0.203 | [−6.883; 1.098] | 0.040 |
| Small 8-Trajectory | Time (m) | 0.292 ± 0.100 | 0.331 ± 0.114 | −0.008 (0.014) | −0.530 | 0.598 | 0.598 | [−0.036; 0.021] | 0.005 | 0.429 ± 0.174 | 0.314 ± 0.108 | 0.114 (0.039) | 2.959 | ***0.005*** | ***0.005*** | [0.037; 0.192] | 0.147 |
|  | Accuracy error (mm) | 8.917 ± 3.370 | 15.25 ± 6.351 | −1.103 (0.471) | −2.341 | ***0.023*** | 0.092 | [−2.050; −0.157] | 0.097 | 7.929 ± 4.009 | 12.536 ± 6.101 | −4.607 (1.277) | −3.608 | ***0.001*** | ***0.001*** | [−7.171; −2.044] | 0.203 |
|  | Work (J) | 16.05 ± 5.445 | 24.906 ± 8.387 | −1.290 (0.725) | −1.780 | 0.081 | 0.162 | [−2.744; 0.165] | 0.058 | 12.962 ± 7.322 | 21.111 ± 8.431 | −8.149 (1.963) | −4.151 | ***<0.001*** | ***<0.001*** | [−12.090; −4.208] | 0.253 |
|  | Velocity (%) | 41.417 ± 10.858 | 43.375 ± 13.411 | 0.630 (1.161) | 0.543 | 0.590 | 0.787 | [−1.701; 2.961] | 0.006 | 30.536 ± 12.691 | 42.536 ± 12.203 | −12.000 (3.146) | −3.814 | ***<0.001*** | ***<0.001*** | [−18.316; −5.684] | 0.222 |
| Large 8-Trajectory | Time (m) | 0.333 ± 0.099 | 0.327 ± 0.080 | −1.731 (1.470) | −1.177 | 0.245 | 0.245 | [−4.684; 1.222] | 0.027 | 0,357 ± 0,117 | 0.330 ± 0.087 | 0.027 (0.028) | 0.957 | 0.343 | 1.372 | [−0.029; 0.082] | 0.018 |
|  | Accuracy error (mm) | 8.500 ± 3.205 | 16.733 ± 7.732 | −2.123 (1.604) | −1.323 | 0.192 | 0.384 | [−5.344; 1.099] | 0.034 | 11.643 ± 5.485 | 13.074 ± 7.343 | −1.501 (1.633) | −0.919 | 0.362 | 0.724 | [−4.780; 1.779] | 0.017 |
|  | Work (J) | 22.815 ± 10.380 | 35.693 ± 9.952 | −2.879 (1.867) | −1.542 | 0.129 | 0.516 | [−6.630; 0.871] | 0.045 | 27.343 ± 11.619 | 19.970 ± 11.893 | −2.669 (3.011) | −0.887 | 0.380 | 0.507 | [−8.716; 3.378] | 0.015 |
|  | Velocity (%) | 49.083 ± 13.853 | 56.733 ± 11.436 | −2.016 (1.666) | −1.210 | 0.232 | 0.309 | [−5.362; 1.330] | 0.028 | 50.607 ± 13.653 | 53.333 ± 12.908 | −2.575 (3.466) | −0.743 | 0.461 | 0.461 | [−9.537; 4.388] | 0.011 |
| Coins | Time (m) | 0.775 ± 0.176 | 0.756 ± 0.261 | −1.999 (1.599) | −1.251 | 0.217 | 0.289 | [−5.208; 1.210] | 0.030 | 0.921 ± 0.274 | 0.764 ± 0.225 | 1.157 (0.064) | 2.466 | ***0.017*** | 0.068 | [0.029; 0.285] | 0.107 |
|  | Accuracy error (mm) | 6.667 ± 3.172 | 11.75 ± 5.106 | −0.993 (0.399) | −2.488 | ***0.016*** | 0.064 | [−1.794; −0.192] | 0.108 | 6.893 ± 3.213 | 9.571 ± 5.014 | −2.679 (1.081) | −2.478 | ***0.017*** | ***0.034*** | [−4.848; −0.509] | 0.107 |
|  | Work (J) | 19.418 ± 10.164 | 31.996 ± 13.592 | −2.691 (1.210) | −2.225 | ***0.031*** | 0.062 | [−5.120; −0.263] | 0.088 | 22.305 ± 11.764 | 26.605 ± 13.598 | −4.301 (3.277) | −1.312 | 0.195 | 0.195 | [−10.880; 2.279] | 0.033 |
|  | Velocity (%) | 22.917 ± 7.513 | 29.00 ± 10.380 | −0.528 (0.864) | −0.611 | 0.544 | 0.544 | [−2.264; 1.207] | 0.007 | 21.107 ± 7.651 | 26.393 ± 9.605 | −5.286 (2.342) | −2.257 | ***0.028*** | ***0.037*** | [−9.988; −0.584] | 0.091 |

For each robotic task, MANOVA models were applied with robotic parameters (Area (m^2^) or Accuracy error (mm), Time (m), Work (J), Velocity (%)) as dependent variables. HFCS level and limb condition (LAS vs MAS) was included as the main clinical factor, while hand dominance and age as additional factors. HFCS group 1: n = 12; HFCS group 2: n = 16; LAS: n = 28; MAS: n = 28. For each variable are reported: descriptive data as mean ± SD, β coefficients (SE), t-values, p-values, FDR-adjusted p-values (p_adj). 95% confidence intervals (CI) and effect sizes expressed as partial eta squared (ηp²) are reported.

**Table S3 - MANOVA results for robotic parameters across tasks according to HFCS levels and limb condition**

|  | **Outcome** | **LAS Mean ± SD** | **MAS Mean ± SD** | **β (SE) MACS level** | **t** | **p** | **p_adj** | **95% CI** | **ηp²** | **β (SE) limb** | **t** | **p** | **p_adj** | **95% CI** | **ηp²** |
| --- | --- | --- | --- | --- | --- | --- | --- | --- | --- | --- | --- | --- | --- | --- | --- |
| Small ROM | Area (m^2^) | 0.027 ± 0.013 | 0.034 ± 0.015 | 0.008 (0.004) | 2.100 | ***0.041*** | 0.082 | [0.000; 0.015] | 0.080 | −0.007 (0.004) | −1.924 | 0.060 | 0.080 | [−0.014; 0.000] | 0.068 |
|  | Time (m) | 0.657 ± 0.169 | 0.625 ± 0.143 | 0.064 (0.041) | 1.544 | 0.129 | 0.172 | [−0.019; 0.147] | 0.045 | 0.032 (0.041) | 0.788 | 0.434 | 0.434 | [−0.050; 0.114] | 0.012 |
|  | Work (J) | 5.32 ± 5.10 | 7.86 ± 5.96 | 3.141 (1.477) | 2.127 | ***0.038*** | 0.152 | [0.176; 6.105] | 0.081 | −2.547 (1.454) | −1.751 | 0.086 | 0.115 | [−5.467; 0.373] | 0.057 |
|  | Velocity (%) | 17.86 ± 5.13 | 22.46 ± 5.35 | 1.513 (1.424) | 1.063 | 0.293 | 0.293 | [−1.345; 4.371] | 0.022 | −4.607 (1.402) | −3.286 | ***0.002*** | ***0.008*** | [−7.422; −1.792] | 0.175 |
| Large ROM | Area (m^2^) | 0.142 ± 0.018 | 0.158 ± 0.025 | 0.016 (0.005) | 3.070 | ***0.003*** | ***0.006*** | [0.006; 0.027] | 0.156 | −0.016 (0.005) | −2.973 | ***0.004*** | ***0.016*** | [−0.026; −0.005] | 0.148 |
|  | Time (m) | 0.971 ± 0.257 | 1.004 ± 0.340 | 0.118 (0.077) | 1.517 | 0.135 | 0.180 | [−0.038; 0.273] | 0.043 | −0.032 (0.076) | −0.421 | 0.675 | 0.675 | [−0.185; 0.121] | 0.003 |
|  | Work (J) | 21.48 ± 10.50 | 28.57 ± 15.83 | 11.327 (3.280) | 3.454 | ***0.001*** | ***0.004*** | [4.743; 17.911] | 0.190 | −7.095 (3.230) | −2.197 | ***0.033*** | 0.066 | [−13.579; 0.611] | 0.086 |
|  | Velocity (%) | 32.04 ± 8.08 | 34.93 ± 7.53 | 0.707 (2.036) | 0.347 | 0.730 | 0.730 | [−3.380; 4.794] | 0.002 | −2.890 (2.005) | −1.443 | 0.155 | 0.207 | [−6.918; 1.132] | 0.039 |
| Small 8-Trajectory | Time (m) | 0.429 ± 0.174 | 0.314 ± 0.108 | 0.043 (0.039) | 1.094 | 0.279 | 0.371 | [−0.035; 0.121] | 0.023 | 0.114 (0.038) | 2.986 | ***0.004*** | ***0.004*** | [0.037; 0.191] | 0.149 |
|  | Accuracy error (mm) | 7.929 ± 4.009 | 12.536 ± 6.101 | 3.083 (1.294) | 2.382 | ***0.021*** | 0.084 | [5.681; 0.100] | 0.100 | −4.607 (1.275) | −3.614 | ***0.001*** | ***0.001*** | [−7.166; −2.048] | 0.204 |
|  | Work (J) | 12.962 ± 7.322 | 21.111 ± 8.431 | 3.770 (1.985) | 1.899 | 0.063 | 0.084 | [−0.215; 7.756] | 0.066 | −8.149 (1.955) | −4.168 | ***<0.001*** | ***<0.001*** | [−12.074; −4.224] | 0.254 |
|  | Velocity (%) | 30.536 ± 12.691 | 42.536 ± 12.203 | −2.719 (3.181) | −0.855 | 0.397 | 0.397 | [−9.105; 3.667] | 0.014 | −12.000 (3.133) | −3.831 | ***<0.001*** | ***<0.001*** | [−18.289; −5.711] | 0.223 |
| Large 8-Trajectory | Time (m) | 0.357 ± 0.117 | 0.330 ± 0.087 | 0.001 (0.028) | 0.023 | 0.982 | 0.983 | [−0.056; 0.057] | 0.000 | 0.026 (0.028) | 0.950 | 0.347 | 1.388 | [−0.029; 0.082] | 0.018 |
|  | Accuracy error (mm) | 11.643 ± 5.485 | 13.074 ± 7.343 | 4.021 (1.660) | 2.422 | ***0.019*** | 0.076 | [0.686; 7.355] | 0.105 | −1.496 (1.628) | −0.919 | 0.363 | 0.726 | [−4.765; 1.774] | 0.017 |
|  | Work (J) | 27.343 ± 11.619 | 19.970 ± 11.893 | 5.623 (3.107) | 1.813 | 0.076 | 0.101 | [−0.607; 11.872] | 0.062 | −2.636 (3.046) | −0.865 | 0.391 | 0.521 | [−8.754; 3.482] | 0.015 |
|  | Velocity (%) | 50.607 ± 13.653 | 53.333 ± 12.908 | 0.799 (3.547) | 0.225 | 0.823 | 1.097 | [−6.325; 7.924] | 0.217 | −2.539 (3.478) | −0.730 | 0.469 | 0.469 | [−9.524; 4.446] | 0.011 |
| Coins | Time (m) | 0.921 ± 0.274 | 0.764 ± 0.225 | 0.047 (0.064) | 0.734 | 0.466 | 0.621 | [−0.081; 0.177] | 0.010 | 0.157 (0.063) | 2.475 | ***0.017*** | 0.068 | [0.285; 0.107] | 0.107 |
|  | Accuracy error (mm) | 6.893 ± 3.213 | 9.571 ± 5.014 | 2.203 (1.121) | 1.966 | 0.055 | 0.220 | [−0.047; 4.452] | 0.070 | −2.679 (1.104) | −2.427 | ***0.019*** | ***0.038*** | [−0.463; 0.104] | 0.104 |
|  | Work (J) | 22.305 ± 11.764 | 26.605 ± 13.598 | 4.630 (3.425) | 1.352 | 0.182 | 0.364 | [−2.245; 11.506] | 0.035 | −4.301 (3.373) | −1.275 | 0.208 | 0.208 | [2.470; 0.031] | 0.031 |
|  | Velocity (%) | 21.107 ± 7.651 | 26.393 ± 9.605 | −0.112 (2.387) | −0.047 | 0.963 | 0.963 | [−4.904; 4.680] | 0.000 | −5.286 (2.351) | −2.249 | ***0.029*** | ***0.039*** | [−0.567; 0.090] | 0.090 |

For each robotic task, MANOVA models were applied with robotic parameters (Area (m^2^) or Accuracy error (mm), Time (m), Work (J), Velocity (%)) as dependent variables. MACS level and limb condition (LAS vs MAS) was included as the main clinical factor, while hand dominance and age as additional factors. MACS level 1: n = 16; MACS level 2: n = 12; LAS: n = 28; MAS: n = 28. For each variable are reported: descriptive data as mean ± SD, β coefficients (SE), t-values, p-values, FDR-adjusted p-values (p_adj). 95% confidence intervals (CI) and effect sizes expressed as partial eta squared (ηp²) are reported.
