# Supplementary figures and images for: Feasibility study of the assessment of upper limb function in children with Unilateral Cerebral Palsy using an end-effector robotic device
Source: J Neuroeng Rehabil. 2026 Mar 19;23:143. doi: 10.1186/s12984-026-01950-7 (PMC13123123; doi:10.1186/s12984-026-01950-7)

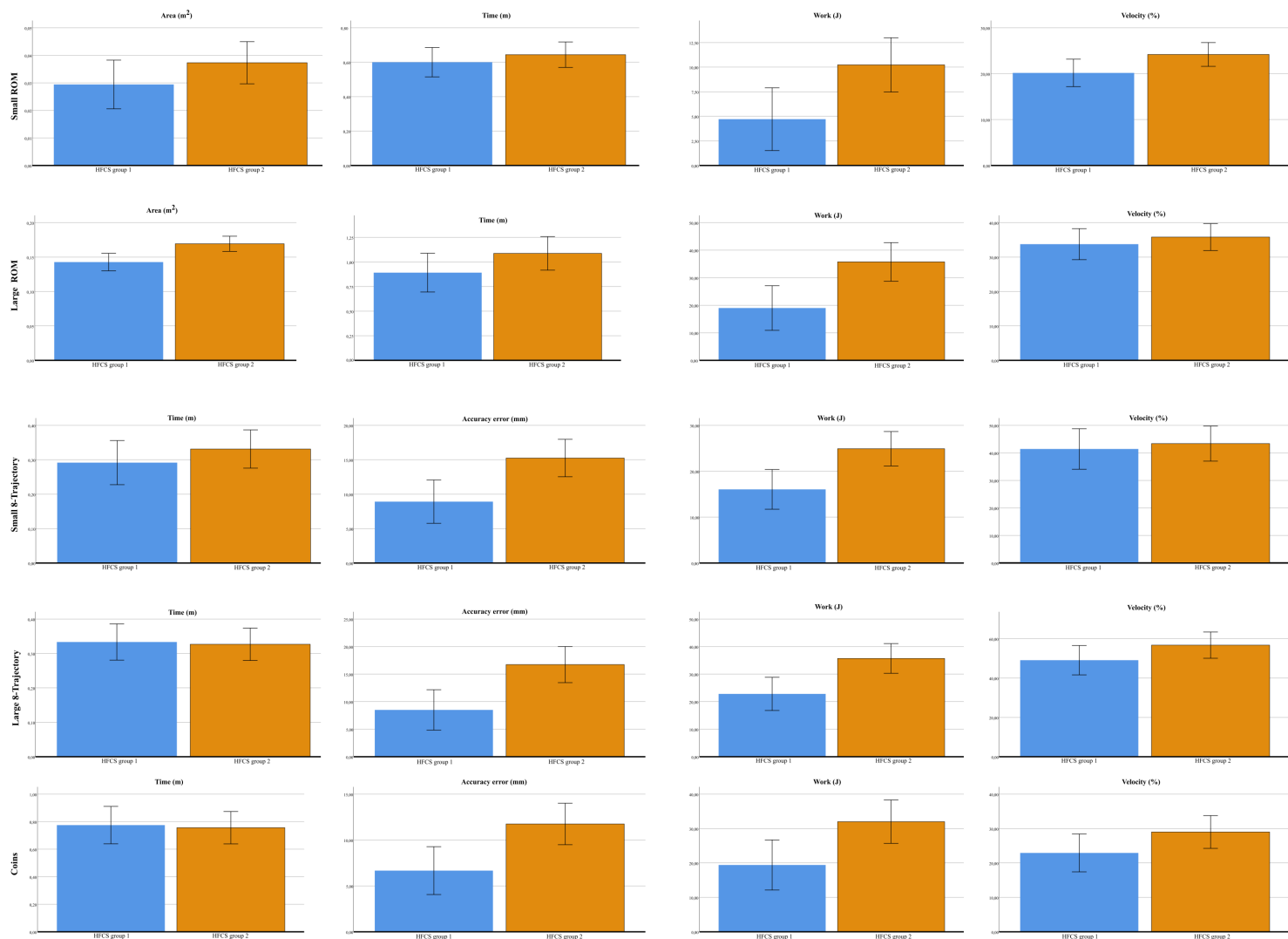

Supplement: Supplementary file 3 — Supplementary Material 3. [file 12984_2026_1950_MOESM3_ESM.pdf]

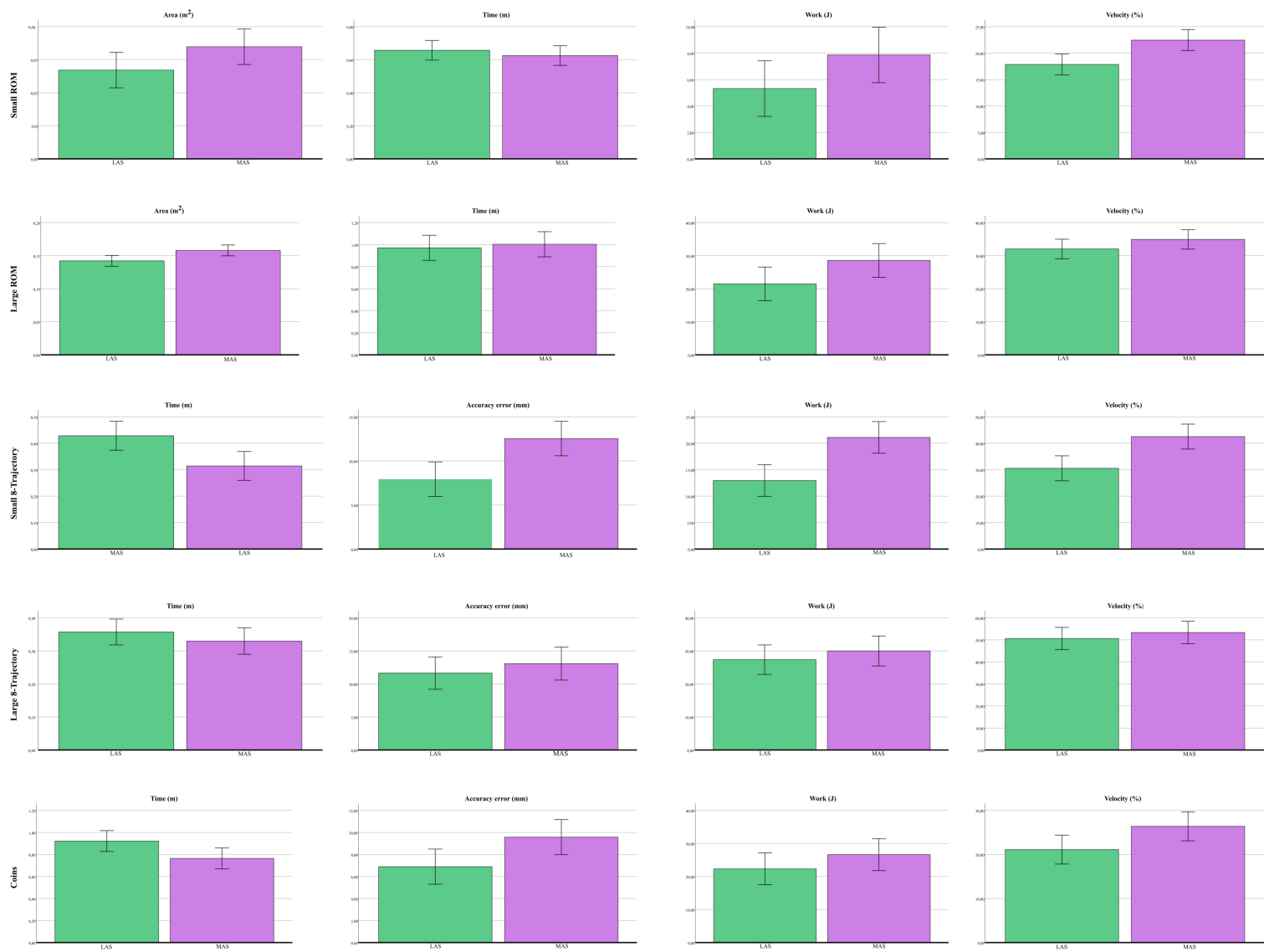

Supplement: Supplementary file 4 — Supplementary Material 4. [file 12984_2026_1950_MOESM4_ESM.pdf]
