# Supplementary material for: Feasibility study of the assessment of upper limb function in children with Unilateral Cerebral Palsy using an end-effector robotic device
Source: J Neuroeng Rehabil. 2026 Mar 19;23:143. doi: 10.1186/s12984-026-01950-7 (PMC13123123; doi:10.1186/s12984-026-01950-7)
Supplement: Supplementary file 5 — Supplementary Material 5. [file 12984_2026_1950_MOESM5_ESM.docx]

**Additional file 1 – Technical description of the robotic system**

*Robotic system configuration*

The study was conducted using the MOTORE robotic system (Humanware, Pisa, Italy), hardware release 4.1 (2020). The device was operated using manufacturer-installed firmware (release 15.8531, 2020) and software suite Motore2020.r3, including GUI (1.6.85b rev. 999), Motapp (1.5.6 GIT 650), Games (1005), and History Viewer (992x998).

Data were acquired at a sampling frequency of 100 Hz. Communication between the robotic system and the controlling interface was primarily wireless via Bluetooth v2.0 Class 2 (2.45 GHz, serial transmission rate 921.6 kBaud); wired communication is also supported by the system architecture.

Task-specific robot settings (e.g., stiffness, viscosity, and other control parameters) were defined in the experimental protocol for each exercise and kept constant across participants.

*Sensing architecture*

The MOTORE system integrates multiple sensing components for planar kinematic and kinetic measurement.

- Force sensing.

Planar interaction forces between the user’s hand and the manipulandum are measured using a two-axis load cell embedded in the handle, pre-amplified and characterized by a reported linearity of 0.2% on full scale (Avizzano et al., 2011). The Force sensitivity reported by manufacturer is < 0.5 N and the Force measurement accuracy is 0.01 N over a full scale of 40 N.

The load cell installed on MOTORE is: Sogein EB 325. Each load cell is released with a full calibration (Certificate of Conformity) including not actuated output (mV), max stroke actuated output (mV) for each axis. These data are available at the manufacturer premises as certificate of calibration for each load cell and thus for each robot. The software takes into account these data. When robot is switched on, a offset correction procedure runs to set the no-load output of the device (considering onboard bit depth and amplification). During the MOTORE conformity check the load cell output is also tested.

- Position sensing.

Planar end-effector position is estimated through an absolute localization system combining:

1. Odometry derived from motor encoders, and

2. An optical localization subsystem based on Anoto® technology, adapted from digital pen applications for real-time planar position tracking over a patterned surface.

An optical CCD sensor positioned beneath the interface case tracks the XY position of the manipulandum relative to a printed reference pattern. An illumination LED ensures adequate lighting conditions for reliable recognition of the observed pattern (Avizzano et al., 2011; Mazzoleni et al., 2018). Position accuracy reported by manufacturer t.

Component-level sensor resolution (e.g., encoder counts or CCD pixel resolution) is not disclosed in publicly available documentation.

Encoder- and optical-based estimates are fused within the device firmware using an Extended Kalman Filter (EKF) (Avizzano et al., 2011).

The optical sensor is calibrated by Anoto®, and the Anoto® Sharp software allows to check the sensor performance once installed in the robot, as reported in the MOTORE Conformity test report.

- Additional sensors and monitoring.

The onboard microcontroller acquires data from an accelerometer via SPI and monitors multiple analog channels. Six 12-bit ADC inputs are used to monitor force sensor signals, battery level, and motor currents (Avizzano et al., 2011).

*Safety limits and safety mechanisms*

Hardware- and software-enforced safety limits are implemented, including:

- Minimum force activation threshold: < 0.5 N
- Maximum exerted force: 50 N (controller-limited)
- Maximum velocity: 0.8 m/s (controller-limited)

Multiple safety mechanisms ensure safe operation:

- Emergency stop buttons: two buttons disable motor power when activated.
- Start-up safety procedure requires release/engagement of the emergency stops to prevent unintended motion at system activation.
- Automatic stop upon handle release: when zero interaction force is detected, the robot stops automatically, acting as a “dead man’s switch”.
- Automatic safety stop: triggered in case of force or velocity limit exceedance, impulsive discharges, or anomalous system behavior.

*Signal processing and filtering*

End-effector position was sampled at 100 Hz and expressed as planar coordinates p(t) = [x(t), y(t)]. The transformation from joint space to Cartesian coordinates is computed in real time by the controller using the system Jacobian.

No additional filtering, smoothing, downsampling, or interpolation was applied by the authors during offline data analysis. All signal conditioning is implemented within the proprietary control architecture of the device.

Specifically:

- An Extended Kalman Filter (EKF) is used internally for state estimation and trajectory tracking. Validation tests at constant speed (0.2 m/s) demonstrated an average positional error below 2 mm (Avizzano et al., 2011).
- On-board analog low-pass filtering is applied to ADC inputs to avoid aliasing. A first-order low-pass filter with a cutoff frequency of 4.2 kHz limits the bandwidth of sensed motor currents (Avizzano et al., 2011).
- Filtering parameters and directionality at the controller level are not user-configurable.

*Trajectory generation and control*

Rehabilitation paths are implemented internally as two-dimensional spline trajectories, reconstructed as cubic C³ splines to ensure continuity of position, velocity, and acceleration and to avoid acceleration discontinuities. Trajectories are implemented in Simulink/Stateflow for basic behaviors and as S-functions for more complex behaviors.

A 2D Newton–Raphson iterative method is used to identify, at each time step, the closest proxy point on the reference trajectory, which directly influences trajectory accuracy metrics. Eight predefined spline geometries with constant minimal curvature are available to ensure algorithmic stability and reduce inertial effects during constant-speed motion (Avizzano et al., 2011).

*Outcome measures*

Outcome measures were computed from the exported kinematic and kinetic signals as follows:

- Area [m²].

Represents the spatial extent of movement during center-out tasks and is computed from planar trajectory points as the maximal radial distance from the center across all movement directions, independent of directional correctness.

To obtain the Covered Area value, the plane is divided into 120 circular sectors (3 degrees each, centered at the center of the plane). For each sector, the maximum distance reached by the patient from the center of the plane is calculated from which the area of the sector is obtained. The covered area is the sum of the areas of all sectors.

This is only considered when the patient is in FREE mode, when the patient moves the robot autonomously.

$$\Delta\theta=\frac{2\pi}{120}=3^{\circ}$$

$$r_{max}\left( \theta_{k} \right)={max}_{i\in k}\parallel p_{i}-p_{0}\parallel$$

$$\boxed{A_{k}=\frac{1}{2}\text{ }r_{max}^{2}\left( \theta_{k} \right)\text{ }\Delta\theta}$$

$$\boxed{A_{covered}=\sum_{k=1}^{120} \frac{1}{2}\text{ }r_{max}^{2}(\theta_{k})\text{ }\Delta\theta}$$

- Accuracy error [mm].

Defined as the mean spatial deviation between the executed trajectory and the ideal reference trajectory, consistent with the minimum-distance definition reported by Mazzoleni et al. (2018).

Accuracy (position error) is defined as the mean spatial deviation between the actual trajectory executed by the participant and the ideal reference trajectory (Mazzoleni et al. 2018).

$${err}_{n}=\sqrt{{(x_{r}-x_{i})}^{2}+{(y_{r}-y_{i})}^{2}}$$

$${acc}_{err}=\frac{1}{N}\sum{err}_{n}$$

Where:

($x_{r},y_{r}$) = real device position

($x_{i},y_{i}$) = ideal position (trajectory)

${err}_{n}$= error at n-th sample

N = number of samples

- Work [Joule].

Mechanical work quantifies the effort exerted by the participant to move the manipulandum along the trajectory and is computed as the line integral of the interaction force along the executed path. The total work both for subject ($W_{P}^{tot}$) and robot ($W_{R}^{tot}$) can be divided into useful ($W_{P}^{use} , W_{R}^{use}$) and unuseful work based on the effort put in place for reaching the target or to perform the task.

$$W_{P}^{tot}=\int_{0}^{T} \left| F_{cell,i} \cdot\hat{u}_{v,i} \right|\parallel u_{v,i}\parallel dt$$

$$W_{P}^{use}=\int_{0}^{T} (F_{cell,i} \cdot\hat{t_{i}})\parallel u_{v,i}^{tan}\parallel dt$$

$$W_{R}^{tot}=\int_{0}^{T} \left| F_{rob,i} \cdot\hat{u}_{v,i} \right|\parallel u_{v,i}\parallel dt$$

$$W_{R}^{use}=\int_{0}^{T} \left| F_{rob,i}^{tan} \cdot\hat{t}_{i} \right|\parallel u_{v,i}^{tan}\parallel dt$$

Where:

$F_{cell,i}$= Force measured by the force cell at discrete time sample $i$

$u_{v,i}$= Velocity vector of the end-effector at discrete time sample $i$

$\hat{u}_{v,i}=\frac{u_{v,i}}{\parallel u_{v,i}\parallel}$= Unit velocity vector

$\hat{t}_{i}$(TVMOB) = Unit tangent vector to spline, expressed in the moving coordinate frame

$u_{v,i}^{\tan}=(u_{v,i}\cdot\hat{t}_{i})\hat{t}_{i}$= Tangential component of the velocity vector along the spline

$F_{rob,i}$= Force exerted by the robot at discrete time sample $i$

$F_{rob,i}^{\tan}=(F_{rob,i}\cdot\hat{t}_{i})\hat{t}_{i}$= Tangential component of the robot force along the spline

$\Delta t$= Sampling period

$N$= Total number of discrete time samples

- Normalized Velocity [%].

Movement speed is reported in normalized form, expressed as a percentage of the user velocity respect to predefined task reference velocity allowed by the device (0.4 m/s).

$$\boxed{v_{\text{norm}}=\frac{v_{\text{user}}}{v_{\text{robot,max}}}}$$

Where:

$$v_{\text{user}}=\text{average user speed}$$

$$v_{\text{robot,max}}=0.4\text{ m/s}$$

No additional post-hoc normalization (e.g., by limb length) was applied.
